# Innovative niosome screening for efficient gene delivery in senescent primary umbilical cord stroma-derived MSCs

**DOI:** 10.1080/10717544.2026.2726650

**Published:** 2026-10-08

**Authors:** Alba Iglesias-Fente, Junquera López-Seijas, María C. Arufe, Juan Antonio Fafián-Labora, Ana Rey-Rico

**Affiliations:** a CICA – Centro Interdisciplinar de Química e Bioloxía, Universidade da Coruña, A Coruña, Spain; b Departamento de Bioloxía, Facultade de Ciencias, Universidade da Coruña, A Coruña, Spain; c Instituto de Investigación Biomédica de A Coruña (INIBIC), Complexo Hospitalario Universitario de A Coruña (CHUAC), Servizo Galego de Saúde (SERGAS), A Coruña, Spain; d Departamento de Medicina, Facultade de Ciencias da Saúde, Universidade da Coruña (UDC), A Coruña, Spain

**Keywords:** Chloroquine, niosome, senescence, transfection, MSCs

## Abstract

The potential of senescent cell clearance to extend healthspan and delay the onset of age-related diseases has driven the development of innovative senotherapeutics. Thus, niosome-based gene therapy has emerged as a promising strategy to overcome the off-target limitations of first-generation senolytics, senomorphics, and senoreverts. In the present study, we investigated two new niosome formulations for genetically modifying primary umbilical cord stroma senescent MSCs (UC-MSCs) in a Palbociclib-induced senescent model, using non-senescent UC-MSCs as a control. These niosomes were formulated by fixing the cationic lipid composition (1,2-di-O-octadecenyl-3 trimethylammonium propane, DOTMA, D) and varying the non-ionic surfactants (polysorbate 20, P20, or polysorbate 80, P80) and the helper lipids (chloroquine, CQ, or cholesterol, CH) (DP20CQ and DP80CH, respectively) at two mole ratios (1:2:1 and 1:2:2 for DP20CQ; 1:2:2 and 1:2:4 for DP80CH). Results on transfection revealed that the DP20CQ formulations outperformed those with DP80CH in both UC-MSCs phenotypes from four different donors. Notably, DP20CQ niosomes prepared at 10/1 and 15/1 DOTMA/DNA ratios achieved the highest transgene expression levels in senescent UC-MSCs, surpassing even the commercial reagent Lipofectamine. Further, DP20CQ formulations exhibited adequate physicochemical properties for transfection and promoted a marked endosomal escape, particularly at the DOTMA/DNA ratio 10/1, without compromising cell viability. These findings underscore the value of niosomes as robust gene delivery vehicles for senescent cells in therapeutic antiaging strategies.

## Graphical abstract

**Figure:**
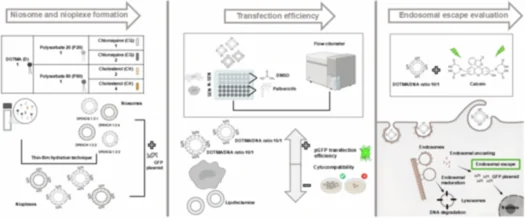

## Introduction

Over the past few years, cellular senescence has gained significant attention on account of its involvement in various physiological and pathological processes (Zhang et al. 2022). Senescent cells are characterised by a stable cycle arrest that limits their proliferative lifespan. However, although this hallmark is crucial, several factors, namely, the DNA damage response, the production of cyclin-dependent kinase inhibitors, such as the cyclin dependent kinase inhibitor 1A (CDKN1A) and the cyclin dependent kinase inhibitor 2A (CDKN2A), and the characteristic senescence-associated secretory phenotype (SASP), which includes chemokines such as C-X-C motif ligand 1 (CXCL1) and interleukin 8 (IL-8), deserve further consideration. Moreover, senescent cells exhibit apoptosis resistance, endoplasmic reticulum stress, nuclear and plasma membrane modifications, and higher activity of senescence-associated *β*-galactosidase (SA-*β*-gal). In addition, the accumulation of old or dysfunctional organelles, such as mitochondria, and biomolecules that lead to the formation of lipofuscin, along with an altered size and a smoother shape, also defines this condition. Despite all changes, senescent cells continue to be metabolically active (Alessio et al. 2019; Calcinotto et al. 2019; Zhang et al. 2022).

The accumulation of senescent cells is one of the main features of aging organisms and can affect regenerative capacities and promote a proinflammatory environment. In this context, senescent cells, together with the decline in the regenerative capacity of the mesenchymal stem cells (MSCs) population (Schultz and Sinclair 2016; Vizoso et al. 2017; Calcinotto et al. 2019), contribute to the development and progression of various age-related diseases, such as osteoarthritis (Malaise et al. 2019) or osteoporosis (Liu et al. 2021), among others (Hernandez-Segura et al. 2018; Calcinotto et al. 2019; Mato-Basalo et al. 2021; Zhang et al. 2022). Despite this, MSCs are among the most studied and applied adult stem cells in regenerative medicine (Vizoso et al. 2017; Jovic et al. 2022; Carballo-Pedrares et al. 2023) with many applications in cell-based therapy (Weng et al. 2022), offering potential benefits in treating these pathologies.

Owing to the healing potential of eliminating senescent cells to extend the healthspan and postpone the development of age-related diseases, the interest in developing new senotherapeutics continues to grow (Zhang et al. 2022). Numerous strategies, including senolytics, which induce senescent cell death; senomorphics, which mitigate the detrimental effects of the SASP without inducing cell death; and senoreverters, which enable senescent cells to re-enter the cell cycle, have been explored. However, the application of senolytics and senomorphics is limited by off-target effects and transient efficacy, while safety concerns remain a limitation when working with senoreverters (Carpenter et al. 2021; Zhang et al. 2022, 2023). Moreover, these pharmacological approaches rely on the systemic modulation of shared survival or inflammatory pathways. Consequently, there is a growing need for more precise therapeutic strategies to modulate cellular senescence (Schmitt et al. 2022).

Based on this, during the past few years, novel techniques, including prodrugs, protein degraders, immunotherapies, and drug (Muñoz-Espín et al. 2018; Popov et al. 2022; Parshad et al., 2025) and gene therapy (López-Seijas et al. 2023), have been taken into consideration to treat this condition (Zhang et al. 2022).

Gene therapy is based on the incorporation of exogenous genetic material into a specific target cell in order to treat a particular disease (Al Qtaish et al. 2020; Vado et al. 2021). Commonly, there are two types of gene carrier systems: viral and non-viral vectors. Of note, non-viral vectors present important advantages when compared to their viral counterparts, as they represent safer alternatives with higher packaging, ease of production, and lower manufacturing costs. However, non-viral vectors are still unable to achieve equal viral transfection efficiencies (Carballo-Pedrares et al. 2023).

Non-viral nanocarriers can be classified into a wide range of nanosized materials, such as cationic lipids, polymers, and inorganic structures (Sainz-Ramos et al. 2021). Regarding cationic lipids, liposomes composed of simple or functional concentric phospholipid bilayer membranes that enclose a hydrophilic core are one of the most popular options when working in medical human treatments (Grijalvo et al. 2019; Attia et al. 2021). Niosomes, in which phospholipids have been replaced by non-ionic surfactants, represent an attractive alternative to these systems as previous studies have reported lower production cost, reduced toxicity, and enhanced stability compared with conventional liposomes (Grijalvo et al. 2019; Attia et al. 2021; Sainz-Ramos et al. 2021; Yasamineh et al. 2022). Moreover, as liposomes, these vectors also contain cationic lipids and ‘helper’ lipids in their structure (Al Qtaish et al. 2020; Sainz-Ramos et al. 2021; Vado et al. 2021).

Focusing on niosomes formulation, non-ionic surfactants (e.g. polysorbates (Tween®) 20 (Villate-Beitia et al. 2018; Gallego et al. 2019) and 80 (Puras et al. 2014; Ojeda et al. 2016; Villate-Beitia et al. 2018; Mashal et al. 2019)) are the responsible molecules to form stable vesicles while avoiding particle aggregation. Cationic lipids (e.g. 1,2-di-O-octadecenyl-3-trimethylammonium propane (DOTMA) (Mashal et al. 2017, 2018; Villate-Beitia et al. 2018; Gallego et al. 2019; Carballo-Pedrares et al. 2021)) have the ability to electrostatically interact with the nucleic acids and the cell membranes because of their positive charge (Grijalvo et al. 2019; Sainz-Ramos et al. 2021). Moreover, these properties strongly influence the transfection efficiency and toxicity of the system. ‘Helper' lipids (e.g. cholesterol (Carballo-Pedrares et al. 2021) and chloroquine (Mashal et al. 2019)) enhance the biophysical properties of the vector, such as stability, permeability, morphology, nucleic acid release, storage time, and fluidity. These lipids also have an impact on the cellular uptake and the internalization mechanisms (Grijalvo et al. 2019; Sainz-Ramos et al. 2021). Therefore, all of these properties depend on the composition of the niosome formulation as well as the production method.

Notably, while many of these studies have explored the use of niosomes to target the retina (Villate-Beitia et al. 2018; Mashal et al. 2019; Gallego et al. 2019), brain (Mashal et al. 2018), and even MSCs (Carballo-Pedrares et al. 2021; Vado et al. 2021; Carballo-Pedrares et al. 2022), only one previous study from our group has probed its potential for genetic modification of senescent MSCs (López-Seijas et al. 2023). However, in that work, the niosomal formulations employed were not optimised in terms of composition and mole ratios, which limited transfection efficiency in this cellular phenotype.

Recent findings have shown that optimizing the composition of niosomal formulations for gene delivery significantly enhances the transfection efficiency of immortalised bone marrow-derived MSCs (López-Seijas et al. 2025). However, whether these optimised systems can be effectively translated to primary senescent umbilical cord-derived MSCs, which exhibit a distinct phenotype, remains unexplored.

Accordingly, the present study evaluated, for the first time, the performance of optimised niosomal formulations in primary senescent UC-MSCs, bridging formulation optimization with a clinically relevant senescent cell model. Niosome formulations were produced by combining the cationic lipid DOTMA (D) with the non-ionic surfactants polysorbate 20 (P20) or 80 (P80) and the helper lipids chloroquine (CQ) or cholesterol (CH) at two different mole ratios (1:2:1 and 1:2:2 for DP20CQ; 1:2:2 and 1:2:4 for DP80CH). The resulting niosome formulations were then combined with a GFP plasmid to obtain nioplexes. Niosomes and nioplexes were characterised in terms of size, zeta potential, polydispersity index (PDI), DNA condensation ability andprotection capacity, and complexation ability. Additionally, GFP expression and cytocompatibility were studied in non-senescent and senescent Palbociclib-induced UC-MSCs derived from four different donors. Finally, the endosomal escape ability of the most suited niosome formulations was explored.

## Materials and methods

### Materials

Polysorbate 20 (P20, MW 1105 g/mol; Cat. No. L15029), polysorbate 80 (P80, MW 1064 g/mol; Cat. No. L13315), and chloroquine (CQ, MW 515.5 g/mol; Cat. No. J64459) were obtained from Alfa Aesar (Kandel, Germany). 1,2-di-O-octadecenyl-3-trimethylammonium propane (DOTMA, D, MW 670.6 g/mol; Cat. No. 890898P) was purchased from Avanti Research (Alabama, USA). Dulbecco’s modified Eagle’s medium (DMEM; Cat. No. 11995073), phosphate-buffered saline (PBS; Cat. No. 10010031), fetal bovine serum (FBS; Cat. No. A5256701), penicillin/streptomycin (P/S, 1000 U/ml; Cat. No. 15140122), trypsin-EDTA (0.25%; Cat. No. 25050014), Opti-MEM^™^ reduced serum medium (Cat. No. 11058021), type II dispase (Cat. No. 17105041) and type I collagenase (Cat. No. 11500536), were obtained from Gibco (New York, USA). Paraformaldehyde (37 wt. %; Cat. No. 252549), Sudan Black B (Cat. No. 199664), crystal violet (Cat. No. C0775), uranyl acetate (Cat. No. 73943), bovine serum albumin (BSA; Cat. No. 422371X), and calcein (Cat. No. C0875) were purchased from Sigma–Aldrich (Darmstadt, Germany). The GreenSafe Premium (Cat. No. MB13201) and NZY Reverse Transcriptase kit (Cat. No. MB12401) were obtained from NZYTech (Lisboa, Portugal), and the PowerUp^TM^ SYBR® Green Master Mix (Cat. No. A25742) from Applied Biosystems (California, USA). Annexin-V-APC (Cat. No. AnxA100) was supplied by MabTag (Oldenburg, Germany) and Palbociclib (Cat. No. HY-50767) from MedChem Express (Sollentuna, Suecia). Tris-Borate-EDTA (TBE buffer; Cat. No. BP13331), sodium dodecyl sulfate (SDS, MW 288.4 g/mol; Cat. No. BP166500), N-2-hydroxyethylpiperazine-*N*-2-ethanesulfonic acid (HEPES, 1 M; Cat. No. 15630056), Triton^TM^ X-100 (Cat. No. BP151100), and agarose (Cat. No. BP1356100) were purchased in Fisher Bioreagents (Pennsylvania, USA). Propidium iodide staining solution (Cat. No. 556463), CD45 FITC (Cat. No. 555489), CD34 PE (Cat. No. 555822), CD90 PECy5 (Cat. No. 554898), CD73 PECy5 (Cat. No. 561260), CD105 FITC (Cat. No. 561443), FITC isotype (Cat. No. 556647), PE isotype (Cat. No. 558595) and PE-Cy5 isotype (Cat. No. 550818) were obtained from BD Biosciences (New York, USA). Lipofectamine® Stem (LPF; Cat. No. STEM00003), DNase I (1000 U; Cat. No. EN0521), DAPI (5 mg/mL; Cat. No. D1306), 5-ethynyl-2’-deoxyuridine (EdU; Cat. No. C10638), fluorescein di-*β*-D-galactopyranoside (FDG; Cat. No. F1179), Alexa Fluor^TM^ 647 (Cat. No. A-21245), Alexa Fluor^TM^ 488 (Cat. No. A-11001), Alexa Fluor^TM^ 647 (Cat. No. A-21235) and SYBR Green Gold (10,000X; Cat. No. S11494) were obtained in Invitrogen (California, USA). Ki67 (Cat. No. 9449) and phosphorylated ribosomal protein (pRPS6) (Cat. No. 4858) were supplied by Cell Signaling Technology (Massachusetts, USA). Acetic acid (Cat. No. AC03431000) was obtained from Scharlau (Barcelona, Spain), and cholesterol 95% (CH, MW 386.7 g/mol; Cat. No. 110190250) and dichloromethane (Cat. No. 348460025) were obtained from Acros Organics (Antwerp, Belgium).

### Niosome preparation

Niosomes were prepared by employing a thin-film hydration technique (Thabet et al. 2022) combining DOTMA with the non-ionic surfactants, polysorbate 20 or polysorbate 80, and the helper lipids, chloroquine (DP20CQ) or cholesterol (DP80CH), respectively, at two different mole ratios (1:2:1 and 1:2:2 for DP20CQ; 1:2:2 and 1:2:4 for DP80CH). All components dissolved in dichloromethane were mixed, and later, the organic solvent was removed under an argon flow. The resultant dried film was resuspended in 2.5 mL of OptiMEM and sonicated at 50 W for 40 s employing a UP200S Sonifier (Hielscher Ultrasound Technology, Teltow, Germany) with a 3 mm probe to obtain the niosome suspension (López-Seijas et al. 2025).

### Plasmid propagation and formation of nioplexes

Plasmid encoding for Green Fluorescent Protein (GFP) (pMC.CMV-MCS-EF1α-GFP-SV40polyA; Cat. No. MN511A-1, System Biosciences, California, USA) was propagated in *E. coli* STLB2, purified using Genopure Plasmid Maxi Kit (Cat. No. 3143414001; Roche, Basel, Switzerland), and quantified following standard procedures.

Nioplexes were obtained by mixing increasing volumes of each niosome suspension with a fixed amount of plasmid (0.304 µg; 250 ng/µL) at DOTMA/DNA mass ratios (w/w) of 5/1, 10/1, and 15/1, equivalent to 2, 5, and 7 nitrogenous (*N*) to phosphorus (*P*) ratios (Carballo-Pedrares et al. 2021). All samples were allowed to interact electrostatically for 30 min at room temperature (RT).

### Physicochemical characterization of niosomes and nioplexes

#### Size and zeta potential

Niosomes and nioplexes size and polydispersity index (PDI) were assessed by Dynamic Light Scattering (DLS) while zeta potential was determined by Electrophoretic Light Scattering (ELS), employing a NanoBrook 90Plus Zeta (Brookhaven Instruments Corporation, New York, USA). In brief, 150 µL of each sample was filtered by 0.45 µm and diluted in 1.5 mL of previously filtered Milli-Q water (dilution 1:10) (López-Seijas et al. 2025). Particle size and zeta potential were calculated by cumulative analysis and electrophoretic mobility employing the Smoluchowski approximation. All measurements were performed in triplicate by employing capillary cuvettes (DTS1070).

#### Agarose gel electrophoresis

The capacity of the niosome formulations to condense and protect DNA against enzymatic degradation was tested by agarose gel electrophoresis assay. To this end, naked GFP (control; DOTMA/DNA ratio of 0) or GFP nioplexes (DOTMA/DNA ratios of 5/1, 10/1, and 15/1; always complexed with 300 ng DNA) were treated with DNase I (0.1 U/µL) for 30 min at 37 °C (López-Seijas et al. 2025). Then, 7% (w/v) SDS solution was added (3 µL/sample), and samples were run on a 0.8% (w/v) agarose gel immersed in TBE for 45 min at 80 V. DNA bands were stained with SYBR Green Gold (0.01% in TBE) and incubated for 1 h under stirring and protected from light. Images were acquired employing the digital Chemi-Doc^TM^ MP Imaging System (Bio-Rad, Madrid, Spain).

#### Evaluation of niosomes complexation ability

Niosomes' capacity to bind and complex DNA was determined by a fluorescence-exclusion titration assay. Briefly, all nioplexes were prepared as mentioned in section ‘Plasmid propagation and formation of nioplexes’, but considering a 50 µL final volume. Then, SYBR Green Gold (200X; 3 μL) was added to each complex and incubated for 10 min at RT protected from light. Finally, 10 mM HEPES was incorporated to obtain a 300 µL final volume to work with a 100 μL/well (López-Seijas et al. 2025). Fluorescence measurements were performed with a Synergy HTX Plate Reader (Agilent, California, USA) in 96-black-well plates (λ_exc_ = 485 nm, λ_em_ = 528 nm). DNA complexation efficiency (%) was expressed as relative fluorescence, normalised to the fluorescence of uncomplexed DNA according to Equation (1):

(1) $$\mathrm{Complexation}\;\mathrm{efficiency}\left(\%\right)=100-\left(F_{\mathrm{sample}}/F_{\mathrm{naked pGPF}}\times 100\right)$$

where *F*_*sample*_ represents the fluorescence intensity of each sample and *F*_*naked pGFP*_ the one recorded for the uncomplexed plasmid.

#### Transmission electron microscopy (TEM)

Niosome dispersions (5 µL) were applied onto carbon-coated grids and left to dry for 5 min. The excess was gently removed using filter paper. All samples were stained with 2% (v/v) uranyl acetate for 30 s and analysed with a high-resolution JEM-1010 TEM (JEOL USA Inc., Massachusetts, USA).

### Umbilical cord stroma MSCs isolation and characterization

MSCs were isolated from caesarean sections performed between June 2023 and May 2024 on healthy women between 26 and 35 years at the Maternity Facility of Complejo Hospitalario Universitario A Coruña (CHUAC). Tissue collection was approved by the supervisor of the Ethical Committee (CEIC: 2019/026) of Galicia, with all patients given written informed consent. All procedures were in conformity with the Helsinki Declaration. In brief, collected tissue was rinsed with PBS, placed in 100 mm culture dishes, cut into explants, and subjected to 5-minute incubations at 37 °C in an enzymatic solution containing 1.2 U/mL type II dispase and 11.2 U/mL type I collagenase. Afterward, MSCs derived from umbilical cord stroma (UC-MSCs) were allowed to attach for 3 days to plastic dishes with DMEM 10% (v/v) FBS and 1% (v/v) P/S (growth medium) before removing the explants. Then, at day 5, the culture medium was filtered using a 0.45 µm cell strainer, and the cells adhered to the plate were detached with trypsin. In this way, we ensured the reseeding of all extracted cells in a new plate with new growth medium. Cells were incubated at 37 °C in a humidified atmosphere containing 5% (v/v) CO_2_ (López-Seijas et al. 2023).

UC-MSCs characterization was performed by flow cytometry (López-Seijas et al. 2023). For this, centrifuge tubes containing 2 × 10^5^ cells were washed and resuspended in PBS with the appropriate dilution (1:100) of the corresponding antibody (Table S2). Cells were incubated for 30 min at RT, protected from light, and under stirring. Subsequently, cells were washed twice by centrifugation with PBS, and the resulting pellets were resuspended in 300 µL of PBS and kept on ice until analysis. Data were generated by a Cytoflex flow cytometer and analysed using Cytoexpert software (Beckman Coulter, Barcelona, Spain).

### Senescence induction in UC-MSCs

Cells were seeded in 48- (transfection assays) or 6-well plates (analysis of the senescent phenotype) at 1.5 × 10^4^ cells/cm^2^ and allowed to adhere for 24 h. Senescence was induced through therapy-induced senescence (TIS), using the compound Palbociclib (CDK4/6 inhibitor) (López-Seijas et al. 2023). Briefly, cells were cultured in growth medium supplemented with Palbociclib at a concentration of 1 µM for 6 days. Non-senescent cells treated for 6 days with growth medium containing DMSO at 1 µM were employed as a negative control.

### Analysis of UC-MSCs senescence phenotype

#### Assessment of senescent UC-MSCs proliferation

Cells were fixed with 4% (v/v) paraformaldehyde for 15 min at RT, followed by three washes with distilled H_2_O. Subsequently, UC-MSCs were stained with a 0.5% (v/v) crystal violet solution for 30 min under stirring and protected from light. Cells were imaged with a microscope (Olympus CKX53 Inverted Led Phase Contrast Microscope, Tokyo, Japan) at 2x magnification (López-Seijas et al. 2023). Quantification of the staining was performed by solubilizing the dye using 30% (v/v) acetic acid for 30 min and measuring the absorbance at 570 nm with a Synergy HTX Plate Reader (Agilent, California, USA).

To specifically address proliferation arrest associated with senescence, DNA synthesis was evaluated using 5-ethynyl-2’-deoxyuridine (EdU) incorporation. Cells were incubated with EdU 10 µM for 24 h prior to fixation and permeabilization. EdU detection was performed according to the manufacturer’s instructions using the Click-iT Plus EdU Imaging Kits (Cat. No. C10638; Invitrogen, California, USA), and nuclei were counterstained with DAPI (300 nM) for 5 min. EdU-stained cells were imaged at 20x magnification using a BioTek Lionheart LX Automated Microscope (Agilent, California, USA). Proliferation arrest was quantified by calculating the percentage of EdU-positive nuclei relative to the total number of DAPI-stained nuclei using ImageJ software.

Moreover, to further characterize the senescent phenotype, immunofluorescence staining for ki67 and phosphorylated ribosomal protein S6 (pRPS6) was performed. Cells were fixed, permeabilised, blocked, and incubated with primary antibodies against ki67 and pRPS6, followed by the corresponding secondary antibodies (Table S2). Nuclei were also counterstained with DAPI and images acquired at 20x magnification using a BioTek Lionheart LX Automated Microscope (Agilent, California, USA) (Alessio et al. 2021).

#### Determination of senescence markers by qPCR

Total RNA was extracted from cells employing E.Z.N.A. ®Total RNA Kit I (Cat. No. R6834-01; Omega Bio-tek, Georgia, USA), following the manufacturer’s protocol. Complementary DNA (cDNA) synthesis was carried out, and qPCR reactions were performed using NZY Reverse Transcriptase and PowerUp^TM^ SYBR® Green Master Mix, respectively, under the supplier’s specifications for primers targeting *CDKN1A*, *CDKN2A*, *CXCL1*, and *IL8*. *ACTB* primers were employed to detect this housekeeping gene for normalization. Amplification and analysis were conducted using a QuantStudio^TM^ 3 (Applied Biosystems, California, USA). Expression levels of the target genes were normalised to *ACTB*, and the relative gene expression was quantified using the 2^−ΔΔCt^ method.

#### Assessment of senescence-associated *β*-galactosidase activity

Cells were incubated with fluorescein di-*β*-D-galactopyranoside (FDG) 50 µM 24 h prior to fixation with 4% (v/v) paraformaldehyde for 15  min at RT. Cells were then washed with 3% (w/v) BSA in PBS and counterstained with DAPI (300 nM) for 5 min. Fluorescence images were acquired at 20X magnification employing a BioTek Lionheart LX Automated Microscope (Agilent, California, USA). SA-*β*-gal fluorescence was quantified using ImageJ software by measuring the integrated signal and expressing the values as pixels/cm^2^.

#### Sudan Black staining

Cells were fixed with 4% (v/v) paraformaldehyde for 15 min at RT and washed with PBS. Then, cells were incubated with Sudan Black B (SBB, 0.7% (w/v) for 2–8 min, protected from light, while monitoring the reaction to ensure optical staining. To remove excess stain, cells were immersed in 50% (v/v) ethanol for 2 min and washed with distilled water three times. Images were captured with a microscope at 10X magnification (Olympus CKX53 Inverted Microscope, Tokyo, Japan). Sudan black staining was quantified as pixels/cm^2^ employing Image J.

### Transfection assays

For transfection experiments, UC-MSCs were cultured in 48-well plates for 6 days in the presence of DMSO (non-senescent UC-MSCs) or Palbociclib (senescent UC-MSCs) as detailed in section ‘Senescence induction in UC-MSCs’ (López-Seijas et al. 2023). Next, the cells were exposed to the nioplexes previously formed ('Plasmid propagation and formation of nioplexes’) by complexing the niosomes with GFP plasmid (300 ng/well) at DOTMA/DNA ratios of 5/1, 10/1, and 15/1 in OptiMEM medium. UC-MSCs cultured in growth medium without nioplexes and cells transfected with LPF (1 µL/well) served as negative and positive controls, respectively. The cells were then incubated for 24 h at 37 °C and 5% CO_2_ before performing the transfection efficiency and cell viability assays.

### Evaluation of transfection efficiency and cell viability

Transfection efficiency and cell viability were evaluated by flow cytometry. Briefly, both the suspended and the adherent cells were combined and resuspended in a staining solution containing Annexin V Alexa Fluor 647 conjugate (1:1000) and Propidium iodide (1:100). This method enabled the evaluation of transfection efficiencies while monitoring apoptosis and necrosis events. Transfection efficiency was expressed as the percentage of GFP-positive cells (%) and normalised to the fluorescence of the negative control as follows in Equation (2):

(2) $$\mathrm{Normali}s\mathrm{ed}\;\mathrm{positive}\;\mathrm{GFP}\;\mathrm{cells}\left(\%\right)=\%F_{\mathrm{sample}}-\%F_{\mathrm{control}}\;$$

where *F*_*sample*_ represents the fluorescence intensity of each sample and *F*_*control*_ the fluorescence recorded for the negative control. Percentages of viable cells (cells; %) were obtained by subtracting from the total number of cells those apoptotic and necrotic, according to Equation (3):

(3) Cells (%)=%Total−(%Apopototic+%Necrotic)

where *%Total* depicts the total number of cells and *%Apoptotic* and *%Necrotic* the apoptotic and necrotic events, respectively.

To provide a comprehensive overview of the transfection efficiencies reached at the different conditions, normalised positive GFP cells (%) across all donors were combined for both phenotypes (non-senescent and senescent UC-MSCs) to generate a transfection ranking based on the median transfection performance of each formulation. The median was used to provide a robust ranking by minimizing the presence of potential outlier values. All formulations (DP20CQ 1:2:1 and 1:2:2; DP80CH 1:2:2 and 1:2:4) and DOTMA/DNA ratios (5/1, 10/1, and 15/1) were included. The resulting ordinal ranking was represented as a heatmap using a color scale, with higher intensities indicating greater transfection performances. Subsequently, once elucidated the optimal niosome formulations and DOTMA/DNA ratios, GFP expression for these niosomes was assessed via fluorescence microscopy. Cells were washed, fixed with paraformaldehyde (4%), permeabilised with Triton X-100 (0.2%), and stained with DAPI (300 nM) for 5 min (López-Seijas et al. 2025). Images were acquired at 10X magnification in a BioTek Lionheart LX Automated Microscope (Agilent, California, USA).

### Endosomal escape evaluation

To test the ability of the most effective niosome formulations to promote endosomal escape during transfection, calcein, a membrane-impermeable fluorophore, was used as a tracer to monitor endosomal stability after niosome uptake (Su et al. 2011). Cells were seeded in a 96 black clear-bottom well plate at a density of 1.5 × 10^4^ cells/cm^2^ and cultured for 6 days in the presence of Palbociclib (senescent UC-MSCs) or DMSO (non-senescent UC-MSCs), as explained in section ‘Senescence induction in UC-MSCs’ (López-Seijas et al. 2023). Calcein (150 μg/mL; 0.24 mM) (Su et al. 2011) was then added to the cells, either alone or in combination with DP20CQ or DP80CH, using niosome quantities corresponding to the DOTMA/DNA ratios that achieved the highest transfection efficiencies without compromising cell viability (DOTMA/DNA ratio 10/1 for both DP20CQ formulations and 15/1 for DP80CH 1:2:2 in non-senescent cells; DOTMA/DNA ratio 15/1 for DP20CQ 1:2:1, 10/1 for DP20CQ 1:2:2 and 5/1 for DP80CH 1:2:1 in senescent cells). Both non-senescent and senescent cells were incubated for 2 h at 37 °C. Following incubation, the plate was fixed and stained with DAPI as detailed in the previous section. Lastly, cells were imaged at 20X magnification employing a BioTek Lionheart LX Automated Microscope (Agilent, California, USA) and intensity calculated as pixels/cm^2^ employing ImageJ.

### Statistical analysis

Transfection and cell viability studies were performed in at least 4 independent donors, with measurements taken in triplicate. Data are expressed as mean ± standard deviation (SD). Statistical analyses were performed using R x64 3.5.1. and RStudio. For datasets involving multiple experimental conditions, ANOVA was applied, followed by Tukey’s post-hoc test, or Kruskal-Wallis and Wilcoxon-Holm when parametric assumptions of normality and homoscedasticity were not met. For all remaining datasets involving direct comparisons between two conditions, a two-tailed Student's t-test was used. *p* < 0.05 was considered statistically significant.

## Results

### Physicochemical characterization of niosomes and nioplexes

#### Size and zeta potential

Niosome formulations (DP20CQ, mole ratios 1:2:1 and 1:2:2; DP80CH, mole ratios 1:2:2 and 1:2:4) were characterised in terms of size (Figure 1A), polydispersity index (PDI; Table S1), and zeta potential (Figure 1A). Illustrative TEM images of the niosome formulations are displayed in Figure S1.

**Figure 1.**
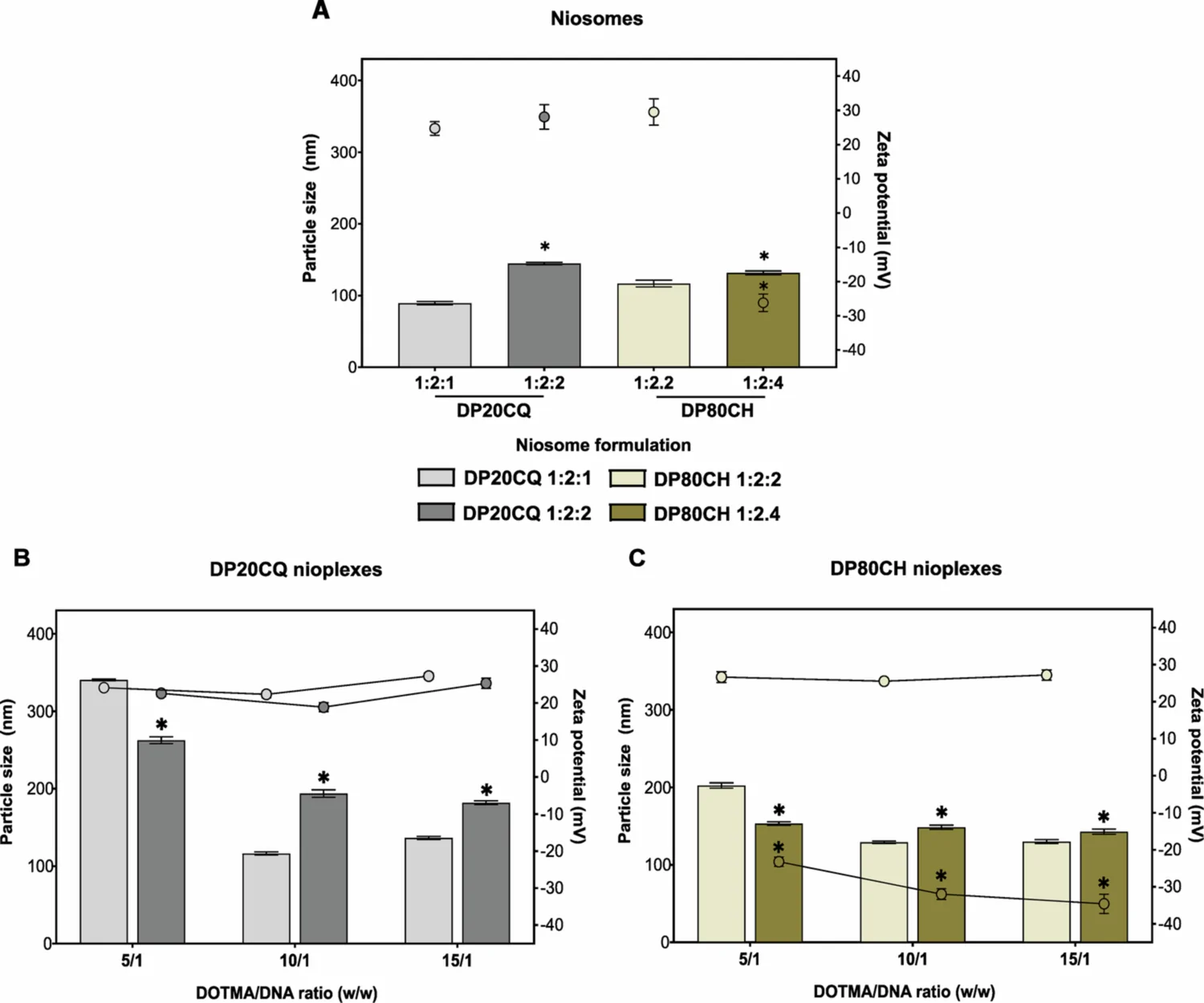
Physicochemical characterization of niosome formulations. Particle size (bars) and zeta potential (symbols and line) of polysorbate 20 and chloroquine (DP20CQ; grey) niosomes at mole ratios 1:2:1 (pale grey) and 1:2:2 (strong grey) and polysorbate 80 and cholesterol (DP80CH; green) niosomes at mole ratios 1:2:2 (pale green) and 1:2:4 (strong green) (A) and their respective complexes after the interaction between DP20CQ (grey) (B) and DP80CH (green) (C) niosomes with the GFP plasmid at DOTMA/DNA ratios of 5/1, 10/1 and 15/1. Data are shown as mean ± SD. * depicts *p* < 0.05, when compared with the lowest mole ratio.

Regarding the size, significant differences were observed among all the formulations studied (*p* < 0.003), being those niosomes with CH more alike than those containing CQ. In both cases, an increase in the mole ratio led to higher niosome sizes (from ~89 nm to ~145 nm in DP20CQ; from ~117 nm to ~132 nm in DP80CH) (Figure 1A). PDI values were around 0.27 in all formulations (*p* < 0.000) (Table S1). Zeta potential measurements were around 27 mV for all niosomes studied, besides those formulated with DP80CH at the highest mole ratio 1:2:4, which showed negative values (−26 mV) and significantly differed from all others (*p* = 0.000) (Figure 1A).

At a subsequent stage, the physicochemical properties of nioplexes resulting from the complexation of niosome formulations with the GFP plasmid at DOTMA/DNA ratios of 5/1, 10/1, and 15/1 were evaluated (size and zeta potential, Figure 1B and C; PDI Table S1).

An overall reduction in size was observed with the increment of the DOTMA/DNA ratio from 5/1 to 10/1 or 15/1 in all formulations explored. Focusing on DP20CQ nioplexes, a significant drop in size at a ratio of 5/1, from ~340 nm in 1:2:1 formulation to ~262 nm in 1:2:2 formulation (*p* = 0.000), was appreciated. The opposite trend was observed for DOTMA/DNA ratios 10/1 and 15/1, where a significant increase (*p* = 0.000) from ~126 nm to ~188 nm was evidenced (Figure 1B). Similar tendencies were observed for DP80CH nioplexes, with a prominent decrease at a ratio of 5/1 between the two formulations, from ~202 nm in 1:2:1 to ~153 nm in 1:2:2 (*p* = 0.000), and a slight increase for ratios of 10/1 and 15/1, from ~129 to ~145 nm (*p* = 0.000) (Figure 1C).

In general, PDI values decreased as the DOTMA/DNA ratio increased, from ~0.30 at 5/1 to ~0.26 at 15/1, although no statistical differences between DOTMA/DNA ratios from each formulation were observed (*p* > 0.103). However, for CH formulation 1:2:4, PDI values for all DOTMA/DNA ratios remained consistently low (~0.23) (*p* > 0.646) (Table S1).

A strong similarity among the zeta potential data of all the DOTMA/DNA ratios was observed in DP20CQ formulations at mole ratios 1:2:1 and 1:2:2 and in DP80CH ones at 1:2:2 mol ratio, with mean values of ~24 mV (Figure 1B and C). A slight decrease was observed in both DP20CQ formulations, as the DOTMA/DNA ratio increased from 5/1 to 10/1, reflected by a decline from ~23 to ~20 mV (*p* > 0.087). Conversely, a significant increase was then observed from ratio 10/1 to 15/1, reaching ~26 mV (*p* < 0.007) (Figure 1B). Electropositivity levels were similar for DP80CH 1:2:2 formulation at all DOTMA/DNA ratios tested, with no statistically significant differences (~27 mV, *p* > 0.927). In contrast, zeta potential for the DP80CH 1:2:4 formulation shifted to negative values, ranging from ~ −23 mV for a 5/1 ratio to ~ −34 mV for 10/1 and 15/1 ratios (*p* = 0.000) (Figure 1C).

#### Evaluation of niosomes complexation ability

The agarose gel electrophoresis assay and the SYBR Green Gold dye exclusion assay were employed to evaluate the ability of the four niosome formulations studied to protect and condense a GFP plasmid (Figure 2, Figure S2).

**Figure 2.**
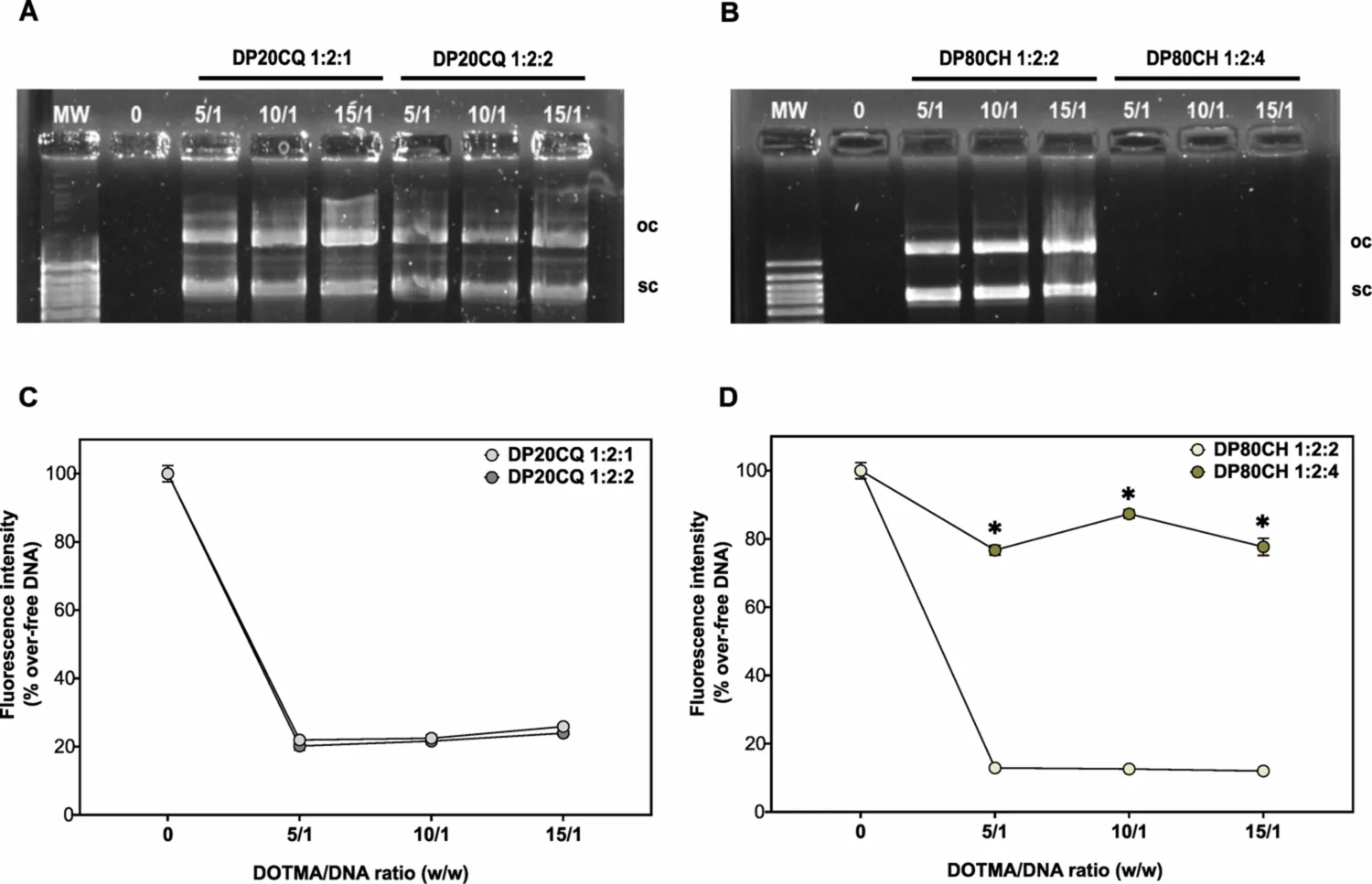
Protection and complexation ability of niosome formulations. DNase protection ability and SDS-induced release visualised by agarose gel electrophoresis of polysorbate 20 and chloroquine (DP20CQ) niosomes mole ratios 1:2:1 and 1:2:2 (A) and polysorbate 80 and cholesterol (DP80CH) formulations mole ratios 1:2:2 and 1:2:4 (B) at DOTMA/DNA ratios of 5/1, 10/1, and 15/1. 0: naked GFP, NC: nicked circular, SC: supercoiled. Complexation ability as fluorescence intensity (% over-free DNA) of DP20CQ (grey) at mole ratios 1:2:1 (pale grey) and 1:2:2 (strong grey) (C) and DP80CH (green) at mole ratios 1:2:2 (pale green) and 1:2:4 (strong green) (D) formulations at DOTMA/DNA ratios of 5/1, 10/1, and 15/1. Data are shown as mean ± SD. * depicts *p* < 0.05, when compared with the lowest mole ratio.

The results from the agarose gel electrophoresis assays in the presence of DNase I are shown in Figure 2A and B. Unlike the results seen with the free plasmid (0), a notable protective effect was observed at all DOTMA/DNA ratios for DP20CQ 1:2:1 and DP20CQ or DP80CH at 1:2:2 mol ratios. These formulations exhibited SC (supercoiled) and OC (open circular) bands after the DNase treatment (Figure 2A and B). In contrast, no DNA protection was demonstrated at any DOTMA/DNA ratios tested from the DP80CH 1:2:4 formulation, as denoted by the absence of detectable DNA bands (Figure 2B).

Regardless of the mole ratio tested, both DP20CQ formulations were able to complex the plasmid (Figure 2C). However, among the DP80CH niosomes formulations, only the one formed at the lowest mole ratio (1:2:2) successfully complexed the GFP plasmid (Figure 2D). Focusing on niosome composition, complexation abilities of DP20CQ nioplexes were around ~77%, and, in general, no statistical differences were noted among DOTMA/DNA ratios inside and across CQ-based formulations (*p* > 0.070) (Figure 2C). DP80CH 1:2:2 niosomes showed ~87% complexation capacity with no statistical differences observed among ratios (*p* > 0.999). Conversely, DP80CH 1:2:4 was the only formulation that exhibited low complexation capacities ~20% as noted by the high fluorescence intensity levels assessed, showing statistically significant differences compared to all other formulations and ratios (*p* < 0.000) (Figure 2D).

### Umbilical cord stroma MSCs characterization

UC-MSCs were characterised by flow cytometry to assess the expression of mesenchymal (CD90, CD73, and CD105) and lack of hematopoietic (CD34 and CD45) markers (Table S2). The analysis revealed high percentages of positivity for mesenchymal markers, with CD90 showing the highest values (60%), whereas the expression of hematopoietic markers remained below 0.3% (Figure S3).

### Analysis of UC-MSCs senescence phenotype

Senescence was characterised by UC-MSCs proliferation capacity (Figure 3A, B, and C and Figure S4), lysosomal activity (Figure 3D, and E and Figure S4), mRNA expression of senescence markers (Figure 3F), and mTOR activation (Figure S4).

**Figure 3.**
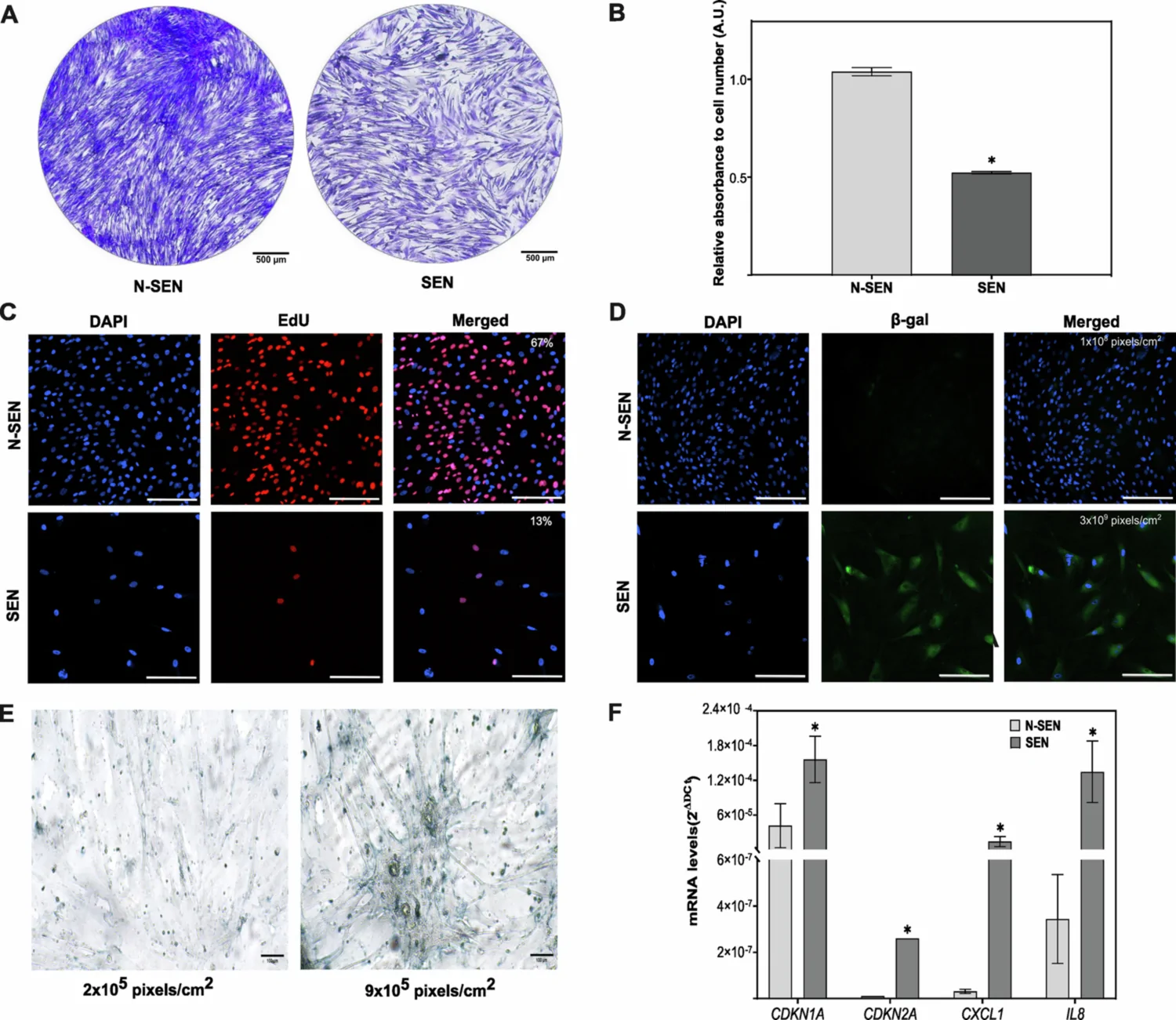
Senescent UC-MSCs characterization. Comparative qualitative (A) and quantitative (relative absorbance to cell number (A.U.)) (B) evaluation of the non-senescent (N-SEN, pale grey) and senescent (SEN, dark grey) proliferation capacity by crystal violet staining. Magnification: 2X. Scale bar: 500 µm. Proliferation arrest evaluated by 5-ethynyl-2’-deoxyuridine (EdU) incorporation, shown qualitatively and quantitatively as the percentage (%) of EdU-positive nuclei, relative to the total number of nuclei. Magnification: 20X. Scale bar: 200 µm. (C). Senescence-associated-*β*-galactosidase (SA-*β*-gal) activity shown as representative fluorescence images and quantified as pixels/cm^2^. Magnification: 20X. Scale bar: 200 µm (D). Lipofuscin production detected qualitatively and quantitatively (pixels/cm^2^) by Sudan Black B staining. Magnification: 10X. Scale bar: 100 µm (E). mRNA levels of senescence markers (*CDKN1A*, *CDKN2A*, *CXCL1*, and *IL8*) in both non-senescent (*N*-SEN, pale grey) and senescent (SEN, dark grey) UC-MSCs (F). Data are shown as mean ± SD. * depicts *p* < 0.05, when compared with the denoted non-senescent phenotype.

A marked reduction in the proliferation capacity was evidenced both qualitatively (Figure 3A) and quantitatively (Relative absorbance to cell number (A.U.), Figure 3B) by crystal violet staining in those UC-MSCs treated with the inhibitor Palbociclib compared to their non-senescent counterparts.

Consistent with proliferation arrest, 5-ethynyl-2’-deoxyuridine (EdU) incorporation revealed a significant decrease in DNA synthesis (*p* = 0.001) in senescent UC-MSCs (13%) compared with non-senescent cells (67%), indicating effective cell cycle arrest associated with senescence (Figure 3C). This finding was further supported by ki67 immunostaining (Figure S4).

Lysosomal alterations associated with senescence were first evaluated by senescence-associated *β*-galactosidase (SA-*β*-gal) activity. Senescent cells exhibited a 24.6-fold increase in SA-*β*-gal signal compared to non-senescent ones, as revealed by fluorescence microscopy and quantitative analysis of signal expressed as pixels/cm^2^ (Figure 3D).

Lipofuscin detection, a waste compound that accumulates due to a decrease in lysosomal activity in senescent cells, was subsequently detected by Sudan Black B staining. The dye intensity, quantified as pixels/cm^2^, showed a 4.3-fold increase in senescent cells compared to the non-senescent ones (Figure 3E).

Quantitative reverse-transcription PCR (qRT-PCR) for markers of cell cycle arrest, such as cyclin-dependent kinase inhibitors (*CDKN1A* and *CDKN2A*), and markers related to the senescence-associated secretory phenotype (SASP), such as C-X-C Motif Chemokine ligand 1 and interleukin 8 (*CXCL1* and *IL8*), were involved to characterize the senescent phenotype in UC-MSCs (Table S3). The upregulation of *CDKN1A*, *CDKN2A, CXCL1*, and *IL8* with fold changes of 1.1, 26.6, 950.4, and 277.4 in senescent cells compared to the non-senescent ones (*p* ≤ 0.048) confirmed the presence of a senescent phenotype for those Palbociclib-treated UC-MSCs (Figure 3F).

In addition, phosphorylated ribosomal protein S6 (pRPS6) immunostaining was detected exclusively in senescent UC-MSCs, consistent with mTOR signaling activation (Figure S4).

### Evaluation of transfection efficiency and cell viability

Transfection efficiencies (Figures 4 and 5; Tables S4 and S5) and cell viability (Figures 8 and 9) of DP20CQ (1:2:1 and 1:2:2) and DP80CH (1:2:2 and 1:2:4) were assessed at 24 h post-treatment in non-senescent and senescent UC-MSCs from four different donors (#1–#4).

**Figure 4.**
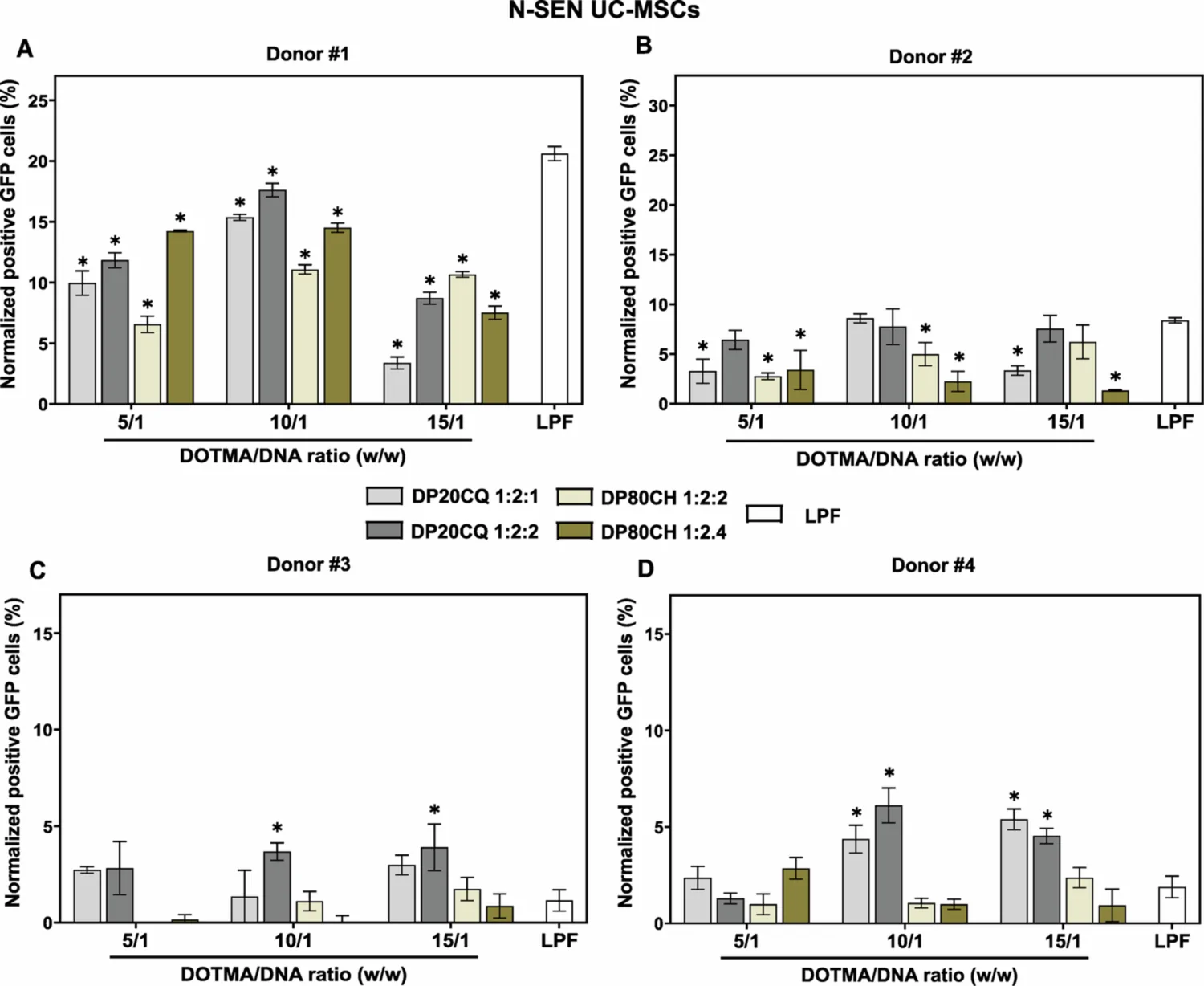
Niosome transfection efficiencies in non-senescent UC-MSCs. Normalised GFP transgene expressions (%) in non-senescent (N-SEN) UC-MSCs from donors #1 (A), #2 (B), #3 (C), and #4 (D) transfected with polysorbate 20 and chloroquine (DP20CQ; grey) niosomes at mole ratios 1:2:1 (pale grey) and 1:2:2 (dark grey) or polysorbate 80 and cholesterol (DP80CH; green) 1:2:2 (pale green) and 1:2:4 (dark green) at DOTMA/DNA ratios 5/1, 10/1 and 15/1. Data are shown as mean ± SD. * depicts *p* < 0.05, when compared with LPF.

**Figure 5.**
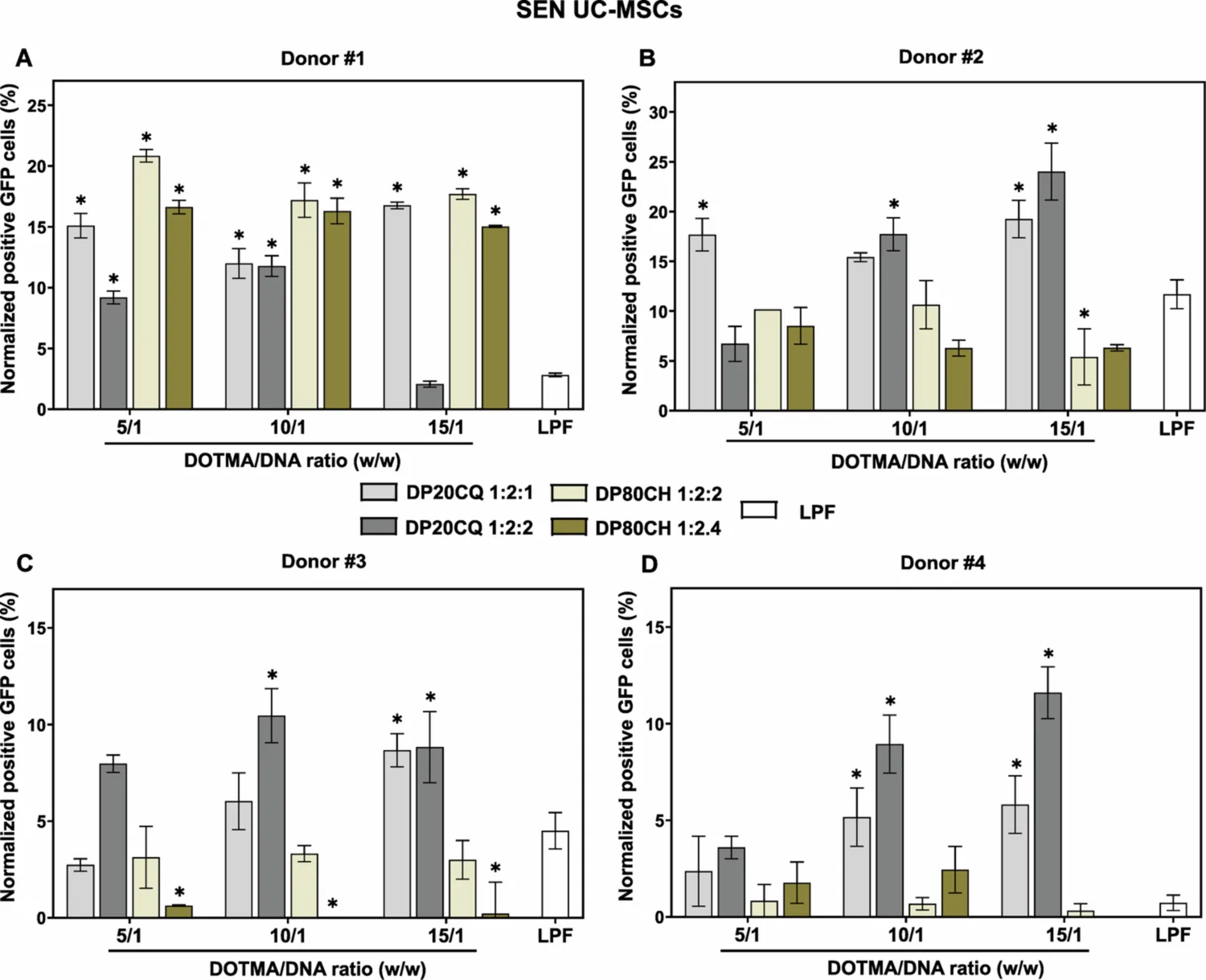
Niosome transfection efficiencies in senescent UC-MSCs. Norrmalised GFP transgene expressions (%) in senescent (SEN) UC-MSCs from donors #1 (A), #2 (B), #3 (C), and #4 (D) transfected with polysorbate 20 and chloroquine (DP20CQ; grey) niosomes at mole ratios 1:2:1 (pale grey) and 1:2:2 (dark grey) or polysorbate 80 and cholesterol (DP80CH; green) 1:2:2 (pale green) and 1:2:4 (dark green) at DOTMA/DNA ratios 5/1, 10/1, and 15/1. Data are shown as the mean ± SD. * depicts *p* < 0.05, when compared with LPF.

Irrespective of the donor and cellular phenotype, analysis of transfection profiles across both non-senescent and senescent UC-MSCs disclosed that DP20CQ formulations always resulted in the highest efficiencies, mostly when using DOTMA/DNA ratios of 10/1 and 15/1.

Focusing on DP20CQ 1:2:1 formulation, for non-senescent cells, a DOTMA/DNA ratio of 10/1 generally produced enhanced transfection effectiveness compared to the 15/1 ratio for donors #1 and #2, although statistically significant differences were only reached in donor #1 (*p* = 0.000) (Figure 4A and B). Conversely, for donors #3 and #4, the 15/1 ratio produced maximal transgene expression levels, although discrepancies compared to the 10/1 ratio did not exhibit a significant effect (*p* > 0.348) (Figure 4C and D). In the case of senescent UC-MSCs, a 15/1 DOTMA/DNA ratio yielded remarkable performance for all donors. Nevertheless, only for donor #1, variations relative to a 10/1 ratio were observed (*p =* 0.000) (Figure 5).

Regarding the DP20CQ 1:2:2 formulation, for non-senescent cells, a DOTMA/DNA ratio of 10/1 broadly resulted in greater transgene expressions (donors #1, #2, and #4) compared to the 15/1 ratio, although differences were only observed for donor #1 (*p* = 0.000) (Figure 4A, B, and D). Exclusively for donor #3, the 15/1 ratio led to enhanced transfection efficiencies, yet this improvement did not reach statistical significance relative to the 10/1 ratio (*p* > 1.000) (Figure 4C). Moreover, when focusing on senescent UC-MSCs, a 15/1 DOTMA/DNA ratio demonstrated marked effectiveness for donors #2 and #4, with discrepancies compared to a 10/1 ratio identified solely in donor #2 (*p* = 0.000) (Figure 5B and D). For donors #1 and #3, a 10/1 ratio showed higher values than 15/1; however, a notable effect was only observed for donor #1 (*p* = 0.000) (Figure 5A and C).

To gain more insight into the performance of DP20CQ niosome formulations, excluding patient- linked variability, a heatmap including all transfection data from the different donors was performed for both non-senescent and senescent cells. This analysis revealed that the highest transgene expression in non-senescent cells was achieved at a DOTMA/DNA ratio of 10/1 for both DP20CQ 1:2:1 and 1:2:2. In contrast, for senescent UC-MSCs, the 15/1 ratio yielded the greatest GFP fluorescence when working with DP20CQ 1:2:1, while the 10/1 ratio was the most effective when employing DP20CQ 1:2:2 formulation (Figure S5, Figure 6, Table S6).

**Figure 6.**
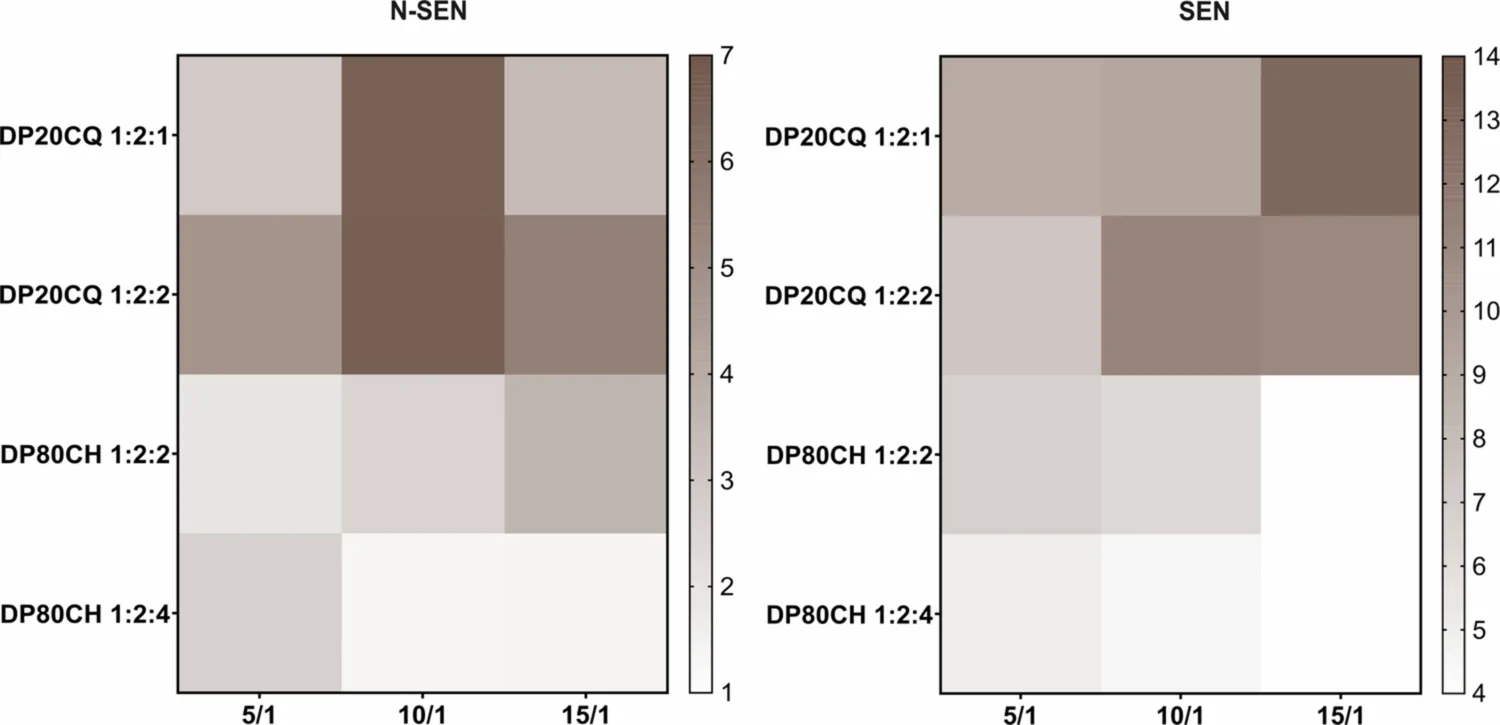
Transfection heatmaps of optimised niosome formulations in UC-MSCs. Transfection efficiency for polysorbate 20 and chloroquine (DP20CQ) niosomes at mole ratios 1:2:1 and 1:2:2 or polysorbate 80 and cholesterol (DP80CH) at mole ratios 1:2:2 and 1:2:4 across DOTMA/DNA ratios of 5/1, 10/1, and 15/1. Data are shown as intensity of colour, where darker and dimmer brown correspond to the highest or lowest values in the transfection ranking number, respectively.

LPF, our positive control, consistently showed lower performance compared to the selected DP20CQ niosome formulations at DOTMA/DNA ratios of 10/1 and 15/1 in both non-senescent (Figure 4) and senescent cells (Figure 5). The only exception was observed in non-senescent cells from donor #1, where LPF achieved significant efficacy compared to the aforementioned formulations (*p* = 0.000) (Figure 4A).

DP80CH formulations demonstrated greater heterogeneity among donors and phenotypes. Hence, while DP80CH 1:2:4 formulation led to maximal gene transfer at a DOTMA/DNA ratio of 5/1 in donors #1 and #4 for non-senescent cells, DP80CH 1:2:2 showed a superior performance for the same phenotype when using a DOTMA/DNA ratio of 15/1 in donors #2 and #3 (Figure 4). For senescent cells, the most favorable results were generally obtained, with DP80CH 1:2:2 at a DOTMA/DNA ratio of 5/1, although statistically significant variation compared to the rest of the ratios inside and across CH-based formulations was only reached in cells from donor #1 (*p* < 0.001). Conversely, the 10/1 ratio of this same formulation achieved the peak GFP expression in senescent cells from donor #2 (Figure 5). As above, a heatmap summarizing data from all donors for both UC-MSCs phenotypes uncovered the most elevated transfection efficiencies for non-senescent cells when using DP80CH 1:2:2 at a DOTMA/DNA ratio of 15/1, while for senescent cells, a 5/1 ratio at the same mole ratio was the one that demonstrated the highest performance (Figure 6). LPF showed stronger (only in non-senescent cells donor #1; *p* = 0.000) or similar gene delivery results compared to DP80CH 1:2:2 at a 15/1 ratio in non-senescent cells (Figure 4) or 5/1 ratio in senescent ones (Figure 5). Only, for senescent cells, DP80CH 1:2:2 5/1 ratio triggered statistically significantly higher levels of GFP expression than LPF (*p* = 0.000) (Figure 5).

To visually validate the heatmap results for the best CQ and CH formulations (Figure 6 and Figure S5), GFP expression was determined by fluorescence microscopy. Images confirmed that DP20CQ niosomes, at both mole ratios studied, outperformed their DP80CH counterparts at the DOTMA/DNA ratios identified as most effective (Figure 7).

**Figure 7.**
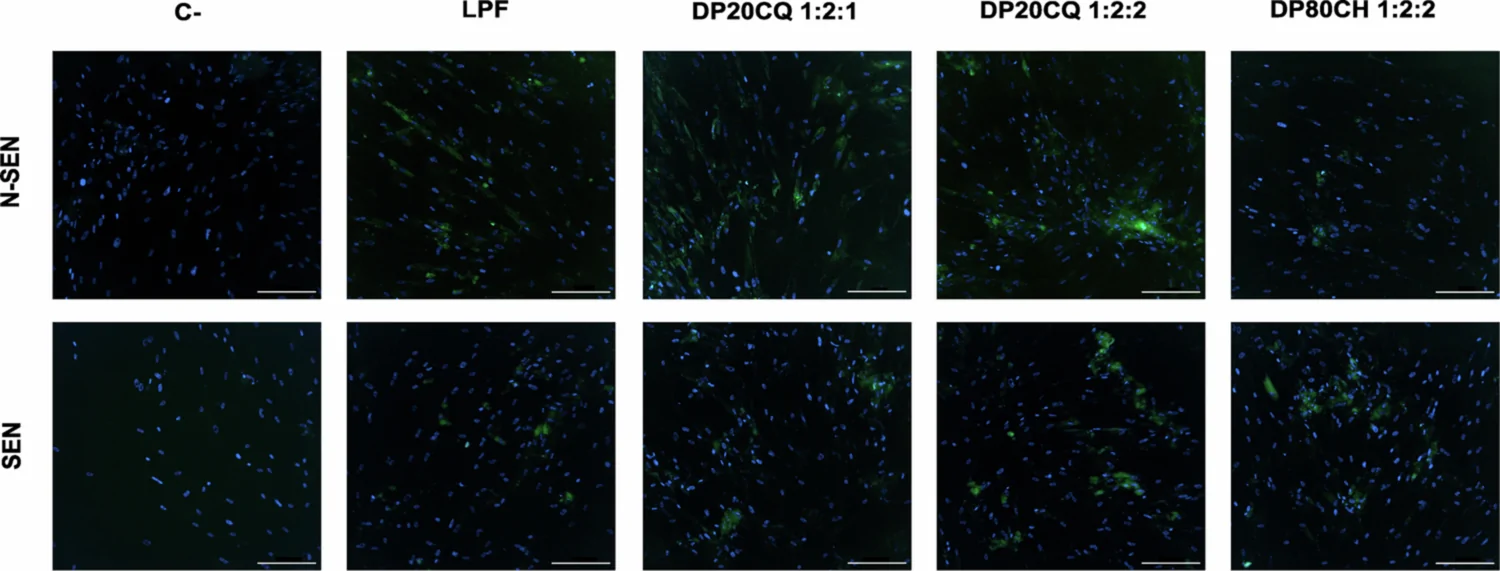
Transfection efficiencies of optimised niosomes in UC-MSCs. GFP and DAPI images of non-senescent and senescent UC-MSCs untransfected (negative control, C-) and transfected with the commercial reagent Lipofectamine (positive control, LPF) or with polysorbate 20 and chloroquine (DP20CQ) niosomes at mole ratios 1:2:1 (DOTMA/DNA ratio 10/1 for non-senescent and 15/1 for senescent cells) and 1:2:2 (DOTMA/DNA ratio 10/1 for non-senescent and senescent cells) or polysorbate 80 and cholesterol (DP80CH) 1:2:2 (DOTMA/DNA ratio 15/1 for non-senescent and 5/1 for senescent cells). Magnification: 10X. Scale bar: 300 µm.

Concerning cell viability, DP20CQ and DP80CH at all mole ratios contributed to great percentages of cell viability for all donors and phenotypes (non-senescent (Figure 8) and senescent cells (Figure 9)) studied, being up to 84%. Transfection of non-senescent cells with DP20CQ and DP80CH nioplexes always led to higher percentages of cell survival than LPF (*p* < 0.015) (Figure 8). A similar trend was observed in senescent cells from donors #1, #2, and #4, showing greater cell viabilities than those cells treated with LPF (*p* < 0.001). Conversely, for donor #3, cells transfected with DP20CQ 1:2:1 at a 10/1 DOTMA/DNA ratio exhibited similar values of cell survival to those reached with the commercial reagent (~84%¸ *p* > 0.999) (Figure 9, Table S6).

**Figure 8.**
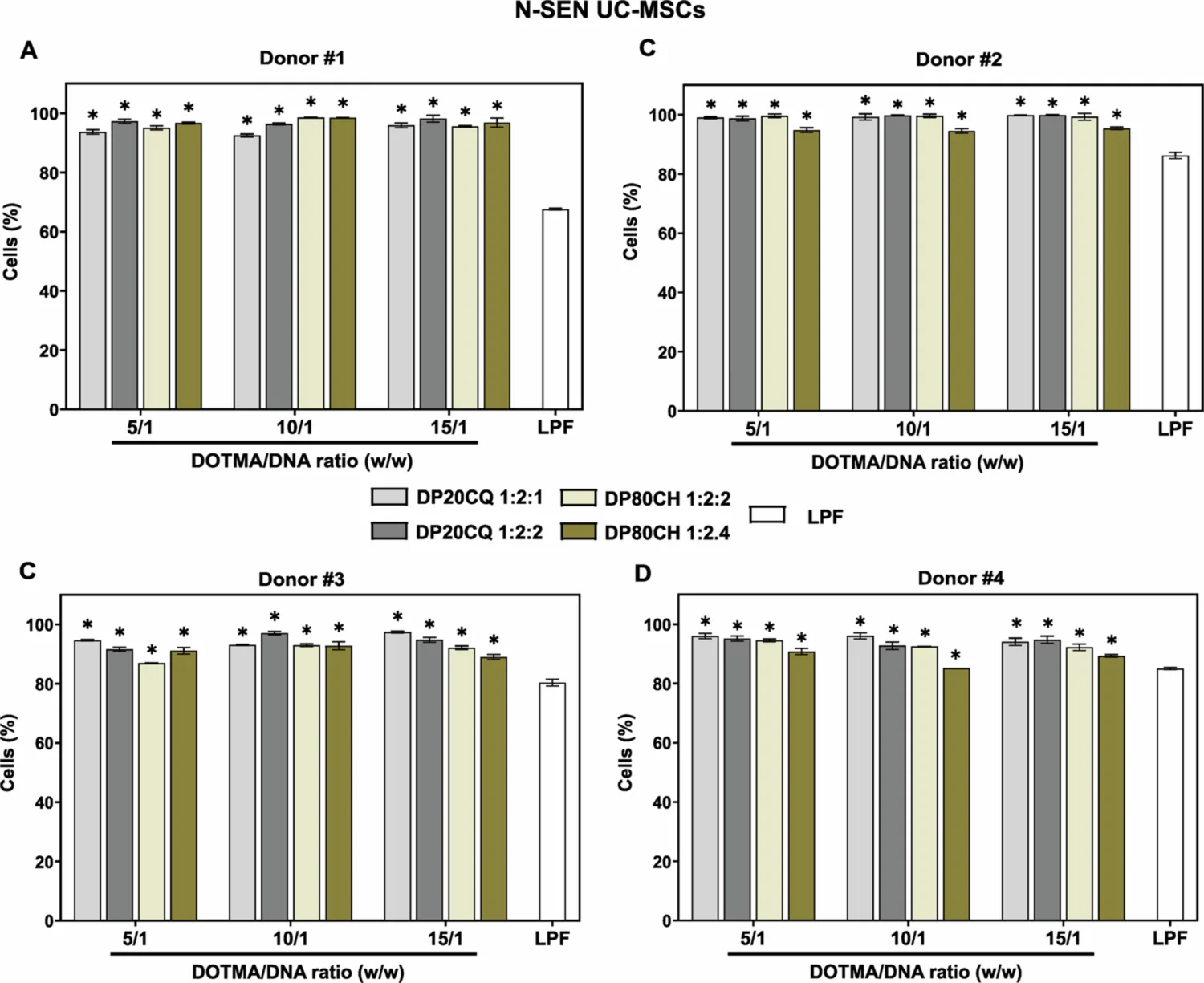
Niosome cell viabilities (%) in non-senescent UC-MSCs. Cell viabilities (%) in non-senescent (NSEN) UC-MSCs from donors #1 (A), #2 (B), #3 (C), and #4 (D) transfected with polysorbate 20 and chloroquine (DP20CQ; grey) niosomes at mole ratios 1:2:1 (pale grey) and 1:2:2 (dark grey) or polysorbate 80 and cholesterol (DP80CH; green) 1:2:2 (pale green) and 1:2:4 (dark green) at DOTMA/DNA ratios 5/1, 10/1 and 15/1. Data are shown as mean ± SD. * depicts *p* < 0.05, when compared with LPF.

**Figure 9.**
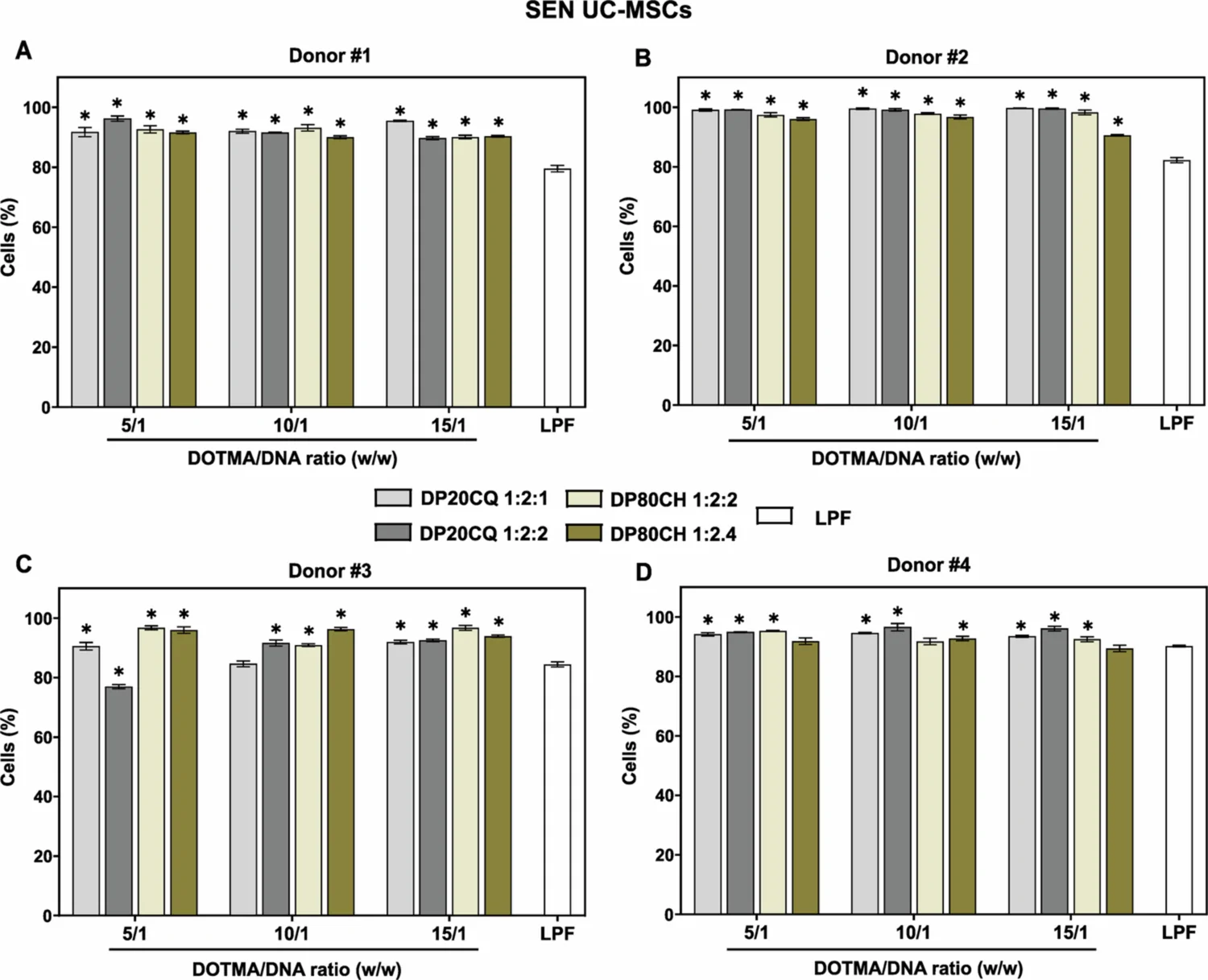
Niosome cell viabilities (%) in senescent UC-MSCs. Cell viabilities (%) in senescent (SEN) UC-MSCs from donors #1 (A), #2 (B), #3 (C), and #4 (D) transfected with polysorbate 20 and chloroquine (DP20CQ; grey) niosomes at mole ratios 1:2:1 (pale grey) and 1:2:2 (dark grey) or polysorbate 80 and cholesterol (DP80CH; green) 1:2:2 (pale green) and 1:2:4 (dark green) at DOTMA/DNA ratios 5/1, 10/1, and 15/1. Data are shown as mean ± SD. * depicts *p* < 0.05, when compared with LPF.

### Endosomal escape evaluation

Calcein was employed as a fluorescent tracer to determine the endosomal escape ability of selected niosome formulations. Based on transfection studies, we chose DP20CQ 1:2:1 and 1:2:2 and DP80CH 1:2:2 at their optimal DOTMA/DNA ratios (DP20CQ 1:2:1: 10/1 and 15/1; DP20CQ 1:2:2: 10/1; DP80CH 1:2:2: 15/1 and 5/1) in non-senescent and senescent cells. Cells without calcein or niosome treatments (C-) and cells exposed to calcein in the absence of niosomes (CC-) were employed as negative controls (Figure 10).

**Figure 10.**
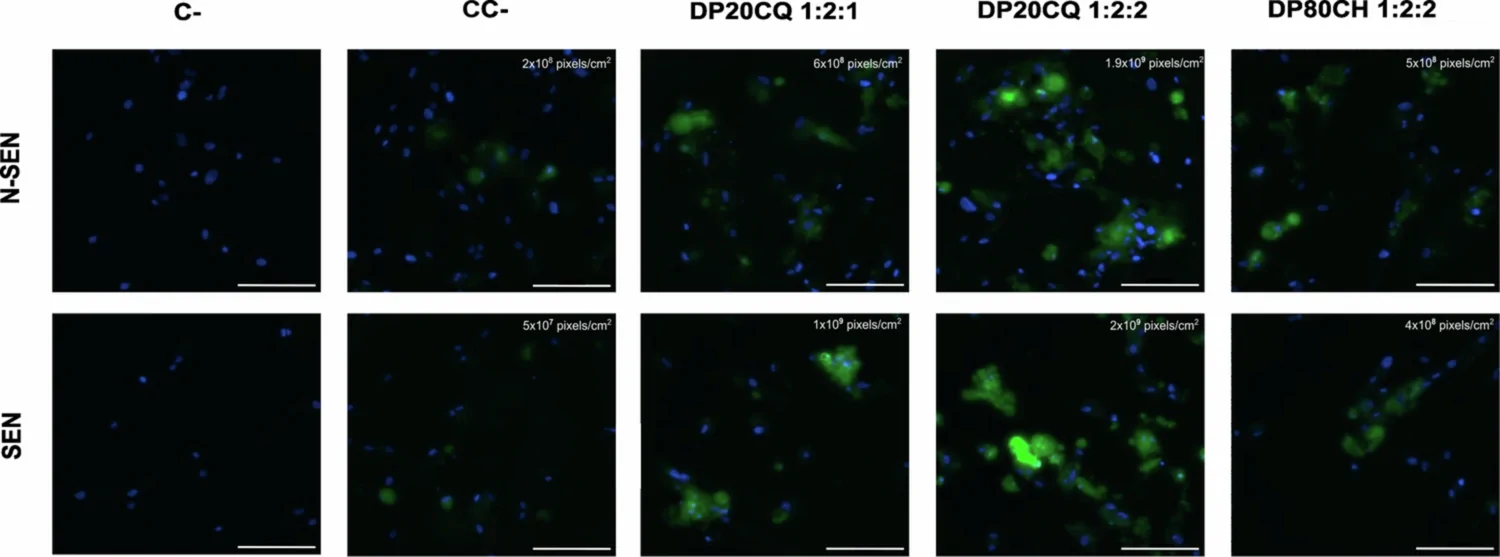
Endosomal escape of optimised niosomes in UC-MSCs. Fluorescent images of non-senescent and senescent UC-MSCs, non-treated (C-) or treated solely with calcein (CC-) or calcein combined with polysorbate 20 and chloroquine (DP20CQ) niosomes at mole ratios 1:2:1 (DOTMA/DNA ratio 10/1 for non-senescent and 15/1 for senescent cells) and 1:2:2 (DOTMA/DNA ratio 10/1 for non-senescent and senescent cells) or polysorbate 80 and cholesterol (DP80CH) at 1:2:2 mol ratio (DOTMA/DNA ratio 15/1 for non-senescent and 5/1 for senescent cells). Quantification of fluorescence intensity is expressed as pixels/cm^2^. Magnification: 20X. Scale bar: 200 µm.

Focusing on non-senescent UC-MSCs, DP20CQ 1:2:2 at a 10/1 ratio revealed a marked endosomal escape, as evidenced by the increase in green fluorescence corresponding to the endosomal disruption and calcein release, compared to DP20CQ and DP80CH 1:2:2 at 10/1 (3.3-fold increase) and 15/1 ratios (3.5-fold increase), respectively. A similar trend was evidenced in senescent cells, exhibiting DP20CQ 1:2:2 formulation at a 10/1 DOTMA/DNA (3.5 and 5.5-fold increase compared to DP20CQ 1:2:1 and DP80CH 1:2:2, respectively), the highest intensities of green fluorescence (Figure 10, Table S6).

## Discussion

Senescent MSCs accumulation is closely linked to the development of aging-related diseases (Hernandez-Segura et al. 2018; Calcinotto et al. 2019; Mato-Basalo et al. 2021; Zhang et al. 2022) and is also associated with the reduction in the regenerative capacity of MSCs (Schultz and Sinclair 2016; Vizoso et al. 2017; Calcinotto et al. 2019). Therefore, the development of new senotherapeutics able to remove this cell population constitutes a potential strategy to extend healthspan and longevity (Zhang et al. 2022). Among the numerous approaches that have been explored, drug (Muñoz‐Espín et al. 2018; Popov et al. 2022; Parshad et al., 2025) and gene therapy (López-Seijas et al. 2023) have emerged as the most promising alternatives capable of avoiding common off-target effects, transient efficacy, and safety concerns associated with senolytics, senomorphics, and senoreverters. Importantly, these alternative strategies enable more precise modulation of senescence than systemic pharmacological interventions (Carpenter et al. 2021; Schmitt et al. 2022; Zhang et al. 2022, 2023).

In light of this, niosomes have arisen as a potential class of non-viral vectors for efficient gene delivery in senescent MSCs (Grijalvo et al. 2019; Attia et al. 2021; Sainz-Ramos et al. 2021; Yasamineh et al. 2022). Although niosomes have been investigated as gene carriers for transfecting MSCs (Carballo-Pedrares et al. 2021; Vado et al. 2021; Carballo-Pedrares et al. 2022), a key population in regenerative medicine (Vizoso et al. 2017; Jovic et al. 2022; Carballo-Pedrares et al. 2023) and cell-based therapy (Weng et al. 2022), only one prior study has examined their potential to genetically modify the senescent phenotype of these cells (López-Seijas et al. 2023). Still, although these results demonstrate the potential of niosomes as novel non-viral nanocarriers for genetic modification of senescent MSCs, the formulations used here were not fully optimised in terms of composition or mole ratios.

By these lines, recent investigations from our group have identified the DP20CQ and DP80CH formulations at 1:2:2 mol- and 5/1 DOTMA/DNA-ratios, as the most efficient for transfecting human bone marrow-derived immortalised non-senescent MSCs. This fact highlights the importance of optimizing niosome composition for effective gene delivery, especially in hard-to-transfect cells such as MSCs, moving niosome-based gene delivery from a trial-and-error approach toward a rational and reproducible methodology (López-Seijas et al. 2025).

Since the cellular model used in the current study, primary senescent umbilical cord-derived MSCs (UC-MSC), exhibits a distinct phenotype, we hypothesised that the optimal mole ratios of niosome-based formulations would differ from those established previously. Senescent UC-MSCs exhibit reduced proliferation, altered membrane composition, and modified endocytic activity, which can affect niosome uptake and gene delivery (Nagano et al. 2021; López-Seijas et al. 2023). To account for these differences and identify formulations that maximize transfection efficiency while preserving cell viability, we systematically tested different ratios of the same core formulations (DP20CQ at 1:2:1 and 1:2:2; DP80CH at 1:2:2 and 1:2:4) in the senescent phenotype. Although the present study was limited to a *therapy-induced senescence model* (TIS), senescent cells are known to share a core set of hallmarks across different senescence-inducing stimuli (Hernandez-Segura et al. 2018; Calcinotto et al. 2019), supporting the identification of a stable senescent phenotype and facilitating its distinction from transient stress-induced states (Alessio et al. 2021). As stimulus-dependent differences may influence nanoparticle uptake and transfection performance, future studies should evaluate the optimised niosomal formulations in additional models of cellular senescence.

All niosomes formulated showed sizes ranging between ~89 and ~145 nm, making them suitable systems for gene delivery applications according to previous literature (Puras et al. 2014; Mashal et al. 2017). Furthermore, in good agreement with previous studies (Ojeda et al. 2016), an increase in the size of niosomes containing CQ was observed as the amount of this helper lipid raised from 1:2:1 to 1:2:2. Irrespective of the formulations studied, the electrostatic interaction between niosomes and the GFP plasmid promoted an efficient DNA condensation (Mashal et al. 2018), as denoted by the reduction in size observed from the DOTMA/DNA ratio of 5/1–15/1.

Focusing on zeta potential, values were approximately 27 mV for DP20CQ at both mole ratios tested and for DP80CH only at the lowest one (1:2:2). It is worth noting that, whereas the electropositivity of DP80CH nioplexes remained relatively constant across all DOTMA/DNA ratios, a decrease in this parameter was observed for DP20CQ nioplexes at lower DOTMA/DNA ratios (5/1 and 10/1), which likely occurred owing to the addition of the GFP plasmid. However, an increment in the zeta potential was evidenced at the highest ratio (15/1) tested, probably as a result of the neutralization of the negatively charged DNA by the cationic lipid (Ojeda et al. 2016; Mashal et al. 2018). In general, these electropositivity values suggest long-lasting stability and homogeneity (Gallego et al. 2019; Mashal et al. 2019), as well as spontaneous electrostatic interactions with DNA, also facilitating their binding to the negatively charged cell membrane prior to cellular uptake (Puras et al. 2014; Mashal et al. 2017; Villate-Beitia et al. 2018). Surprisingly, the DP80CH 1:2:4 formulation exhibited negative zeta potential values for both niosomes and nioplexes, making them less suitable to establish electrostatic interactions with DNA (Puras et al. 2014; Mashal et al. 2017; Villate-Beitia et al. 2018) but enhancing their capacity to avoid particle aggregation (Cao et al. 2018; Liu et al. 2022). This negative charge could be associated with the increased negative surface potential of niosomes, resulting from the higher cholesterol concentration. One possible explanation is the asymmetric polarity of the cholesterol hydroxyl group (Ahmadi et al. 2024), in which the oxygen atom exhibits a partial negative charge and the hydrogen atom has a partial positive charge. In a buffered medium such as OptiMEM, this may facilitate interactions between charged ions such as phosphates and the niosomal membrane, potentially contributing to the overall negative zeta potential (Manosroi et al. 2010). Furthermore, cholesterol has been reported to modulate membrane electrostatics by shifting the potential above the membrane surface towards more negative values, which could effectively reduce the effective exposure of the cationic lipid DOTMA at the shear plane (Doktorova et al. 2017). However, these mechanisms remain hypothetical in the context of the present study and would require further investigation to be confirmed

When studying PDI values, all niosomes and nioplexes formed at the different ratios ranged from ~0.23 to ~0.30, indicating narrow size distributions (Villate-Beitia et al. 2018), which are favorable for gene delivery applications (Mashal et al. 2019).

Additionally, in consistency with the observations reported by Villate-Beitia et al. (Villate-Beitia et al. 2018) and Mashal et al. (Mashal et al. 2018), TEM images revealed spherical nanosystems in DP80CH (1:2:2 and 1:2:4 mol ratios) formulations. However, for the DP20CQ (1:2:1 and 1:2:2 mol ratios) formulations, non-perfectly spherical particles were observed. As the morphology of nanocomplexes plays a critical role in transfection performance, with perfectly spherical particles internalizing more slowly (Attia et al. 2018), these findings may help explain the higher transgene expressions achieved with the CQ-based niosomes.

The ability of nioplexes to condense, protect, and release a DNA plasmid at different DOTMA/DNA ratios was evaluated by SYBR Green Gold dye assay and agarose gel electrophoresis. In consonance with the registered zeta potential values, all formulations successfully condensed and protected DNA against degradation, being an essential factor during transfection (Mashal et al. 2017; Villate-Beitia et al. 2018, Mashal et al. 2019). However, an exception was observed for the DP80CH formulation at a 1:2:4 mol ratio, where the complexation capacity was ~20% and no DNA protection capability was evidenced, as indicated by the absence of SC and OC bands (López-Seijas et al. 2023).

The data from this study indicated that, regardless of the cell phenotype, the highest transfection efficiencies were always achieved with DP20CQ niosomes. Nonetheless, as a result of the inherent variability associated with primary cultures (Ding et al. 2023), together with the intrinsic biological heterogeneity described for MSCs (Alessio et al. 2020) and the challenge that represents the intracellular delivery in UC-MSCs (Yang et al. 2022), no clear differences in terms of transgene expression were detected between the DP20CQ 1:2:1 and 1:2:2 formulations. Importantly, the transfection efficiencies achieved with these optimised niosome formulations were notably higher than those obtained in López-Seijas et al. (López-Seijas et al. (2023)), demonstrating that systematic optimization, rather than enhancement strategies alone, is critical for improving gene delivery in senescent MSCs. While these results were obtained using a GFP reporter plasmid as a proof of concept, they demonstrated the feasibility of gene delivery using optimised niosomal formulations in senescent MSCs. Future studies employing therapeutically relevant cargos will be required to determine whether the transfection efficiencies achieved with the reporter gene can be reproduced with therapeutic transgenes and translated into meaningful therapeutic outcomes.

We previously observed a preferential internalization of niosomes by macropinocytosis in senescent UC-MSCs (López-Seijas et al. 2023). In senescence, this route may be further enhanced by the overexpression of the lymphocyte antigen 6 complex, which induces macropinocytosis as a cell survival mechanism, while there is loss of clathrin- and caveolin-dependent endocytosis (Shin et al. 2020; Nagano et al. 2021).

Since lysosomal-based pathways constitute the primary vesicular transport routes in UC-MSCs (Yang et al. 2022), the existence of vesicle evasion mechanisms is crucial to enhance nanoparticle intracellular delivery in these hard-to-transfect cells (Ding et al. 2023). Alongside, previous studies have shown a reduced cytoplasmic diffusion and endosomal escape ability in primary cells compared with those in cell lines (Yang et al. 2022). Based on this, in order to investigate the enhanced transfection results observed for the CQ niosomes, endosomal escape assays with calcein fluorophore were conducted. Consistent with the known ability of the CQ compound to promote endosomal escape (López-Seijas et al. 2023; Qiu et al. 2023) through the proton sponge effect, DP20CQ niosomes were able to promote efficient endosomal escape, leading to higher transgene expressions, particularly at the major CQ content (1:2:2 mol ratio). This behaviour is consistent with the lysosomotropic nature of CQ, which accumulates within acidic endosomal compartments and buffers the luminal environment. This localised increase in pH drives the continuous activation of endosomal proton pumps, triggered by the chelation of protons with CQ amino groups. The resulting influx leads to a compensatory accumulation of chloride ions and water, creating an osmotic imbalance that results in endosomal swelling and rupture, thereby enabling the release of nanocarriers into the cytosol (Degors et al. 2019; Ding et al. 2023; Pandey and Harris 2023). However, because both the formulation composition and the DOTMA/DNA ratio were varied simultaneously, further studies will be required to elucidate the individual contribution of each parameter to the observed endosomal escape.

In the case of CH-based niosomes, the highest transgene expression levels for both non-senescent and senescent cells were obtained with the lowest mole ratio 1:2:1, in concordance with the absence of condensation and protection capacity observed for ratio 1:2:4. However, the overall lower performance of these niosomes compared to the previously described DP20CQ formulations may be attributed to their reduced endosomal escape capability, as cholesterol does not provide a lysosomotropic contribution comparable to chloroquine. Here, the acidic environment and enzymatic activity within the endosomes may contribute to the degradation of CH-based niosomes, preventing DNA molecules from reaching the nucleus (Villate-Beitia et al. 2018; Ding et al. 2023).

Despite the differences in transfection efficiency, all niosome formulations maintained consistently high viabilities, with DP20CQ formulations regularly showing viabilities above 84%. These formulations demonstrated equal or superior cytocompatibilities compared to the commercial reagent LPF. These results are consistent with previous ones obtained using the same formulations in non-senescent iMSCs (López-Seijas et al. 2025).

## Conclusion

The present study demonstrates the potential of DOTMA, polysorbate 20, and chloroquine (DP20CQ) niosomes formulated at 1:2:1 and 1:2:2 mol ratios and DOTMA/DNA ratios of 15/1 and 10/1 to promote high levels of transgene expression in senescent UC-MSCs without compromising cell viability. Of note, these nanocarriers outperformed the commercial reagent LPF in terms of transfection efficiency. These succeeding results can be attributed to chloroquine’s endosomal escape capacity, through the proton-sponge effect, especially for DP20CQ 1:2:2 formulation at a 10/1 ratio. This fact is especially relevant when designing gene delivery systems for hard-to-transfect cells, such as MSCs. Altogether, these findings establish optimised niosomal formulations as a robust and versatile non-viral genedelivery platform for senescent cells, providing a foundation for future therapeutic strategies aimed at modulating cellular senescence in degenerative conditions such as osteoarthritis. Future studies employing relevant therapeutically relevant senotherapeutic cargos will be necessary to evaluate their therapeutic efficacy, safety, and translational potential.

## Supplementary Material

Supplementary Material Clean copy of Supplementary_data_amended UDRD-2026-0087.R2.docx

## Data Availability

Data will be made available on request.
