# Supplementary material for: Innovative niosome screening for efficient gene delivery in senescent primary umbilical cord stroma-derived MSCs
Source: Drug Deliv. 2026 Oct 8;33(1):2726650. doi: 10.1080/10717544.2026.2726650 (PMC13651833; doi:10.1080/10717544.2026.2726650)
Supplement: Supplementary file 1 [file IDRD_A_2726650_SM2879.docx]

**Supplementary data**

Original research

Iglesias-Fente *et al*

**Innovative niosome screening for efficient gene delivery in senescent primary umbilical cord stroma-derived MSCs**

Alba Iglesias-Fente^1,2^

Junquera López-Seijas^1,2^

María C. Arufe ^1,3^

Juan Antonio Fafián-Labora^1,3^

Ana Rey-Rico^1,2^

^1^CICA - Centro Interdisciplinar de Química e Bioloxía, Universidade da Coruña, 15071 A Coruña, Spain; ^2^Departamento de Bioloxía, Facultade de Ciencias, Universidade da Coruña, 15071 A Coruña, Spain; ^3^Instituto de Investigación Biomédica de A Coruña (INIBIC), Complexo Hospitalario Universitario de A Coruña (CHUAC), Servizo Galego de Saúde (SERGAS) 15006 A Coruña, Spain, Departamento de Fisioterapia, Medicina e Ciencias Biomédicas, Facultade de Ciencias da Saúde, Universidade da Coruña (UDC), Campus de Elviña, 15071 A Coruña, Spain.

Correspondence: Ana Rey-Rico /Juan A. Fafián-Labora

CICA - Centro Interdisciplinar de Química e Bioloxía, As Carballeiras, s/n, Campus de Elviña, Universidade da Coruña, 15071 A Coruña, Spain

Tel: 881 01 5543/ 881 01 5544

Email: [ana.rey.rico@udc.es](mailto:ana.rey.rico@udc.es) / [juan.labora@udc.es](mailto:juan.labora@udc.es)

**Figure S1.** Representative TEM images of niosomes composed of polysorbate 20 and chloroquine (DP20CQ) at 1:2:1 (**A**) and 1:2:2 mole ratios (**B**), and polysorbate 80 and cholesterol (DP80CH) at 1:2:2 (**C**) and 1:2:4 (**D**) mole ratios. Magnification: 200 kX. Scale: 100 nm.

**Figure S2.** Original Image for **Fig 2A** (**A**) and **2B** (**B**).

**Figure S3.** Characterization of UC-MSCS in terms of events from a single donor included in the study considering antibodies CD90 PECy5 and CD73 PECy5 detected by laser B690 (**A**); CD105 FITC and CD45 FITC detected by laser B525 (**B**) and CD34 PE detected by laser B585 (**C**) are presented to illustrate the typical cellular profile.

**Figure S4.** Representative images showing the combined assessment of senescence-associated-β-galactosidase (SA-β-gal) activity, ki67 and phosphorylated ribosomal protein S6 (pRPS6). Panels show SA-β-gal (green)/ ki67 (red) (**A**), SA-β-gal (green)/ pRPS6 (red) (**B**) and Ki67 (green)/pRPS6 (red) (**C**). Magnification: 20X. Scale bar: 200 µm.

**Figure S5.** Scatter plots of optimized niosome formulations in UC-MSCs. Transfection efficiency for polysorbate 20 and chloroquine (DP20CQ) niosomes at mole ratios 1:2:1 and 1:2:2 or polysorbate 80 and cholesterol (DP80CH) at mole ratios 1:2:2 and 1:2:4 across DOTMA/DNA ratios of 5/1 (denoted by triangles), 10/1(denoted by circles) and 15/1 (denoted by inverse triangles). Individual donor values are shown, with each donor represented by a different color (donor 1: red; donor 2: blue; donor 3: green and donor 4: gold). Horizontal bars indicate the mean.

**Table S1.** Polydispersity index (PDI) of polysorbate 20 and chloroquine (DP20CQ) (**left panel**) or polysorbate 80 and cholesterol (DP80CH) (**right panel**) niosomes and nioplexes at DOTMA/DNA ratios 5/1, 10/1 and 15/1. Data are shown as mean ± SD.

| Formulation | Ratio | PDI |
| --- | --- | --- |
| DP20CQ 1:2:1 | 0 | 0.263±0.010 |
|  | 5/1 | 0.302±0.011 |
|  | 10/1 | 0.269±0.040 |
|  | 15/1 | 0.271±0.007 |
| DP20CQ 1:2:2 | 0 | 0.301±0.007 |
|  | 5/1 | 0.306±0.017 |
|  | 10/1 | 0.286±0.014 |
|  | 15/1 | 0.259±0.005 |

| Formulation | Ratio | PDI |
| --- | --- | --- |
| DP80CH 1:2:2 | 0 | 0.270±0.007 |
|  | 5/1 | 0.297±0.011 |
|  | 10/1 | 0.257±0.007 |
|  | 15/1 | 0.245±0.011 |
| DP80CH 1:2:4 | 0 | 0.242±0.011 |
|  | 5/1 | 0.219±0.012 |
|  | 10/1 | 0.245±0.007 |
|  | 15/1 | 0.221±0.006 |

**Table S2.** List of antibodies.

| Antibody | Working dilution | Reference | Company |
| --- | --- | --- | --- |
| Annexin-V-APC | 1:1000 | AnxA100 | MabTag |
| CD90 PECy5 | 1:100 | 554898 | BD Biosciences |
| CD73 PeCy5 | 1:100 | 561260 | BD Biosciences |
| CD105 FITC | 1:100 | 561443 | BD Biosciences |
| CD34 PE | 1:100 | 555822 | BD Biosciences |
| CD45 FITC | 1:100 | 555489 | BD Biosciences |
| Isotype FITC | 1:100 | 556647 | BD Biosciences |
| Isotype PE | 1:100 | 558595 | BD Biosciences |
| Isotype PECy5 | 1:100 | 550818 | BD Biosciences |
| Ki67 | 1:1000 | 9449 | Cell Signaling Technology |
| pRPS6 | 1.200 | 4858 | Cell Signaling Technology |
| Alexa Fluor^TM^ 647 | 1:1000 | A-21245 | Invitrogen |
| Alexa Fluor^TM^ 488 | 1:1000 | A-11001 | Invitrogen |
| Alexa Fluor^TM^ 647 | 1:20 | A-21235 | Invitrogen |

**Table S3.** List of primers sequence for qPCR from R.Costoya (A Coruña, Spain).

| Target | Forward | Reverse |
| --- | --- | --- |
| *CDKN1A* (p21CIP1) | CCAGTCCATTTGATCAGCGG | ACATCTTTCTCCCGGGTCTG |
| *CDKN2A* (p16INK4a) | CGGTCGGAGGCCGATCCAG | GCGCCGTGGAGCAGCAGCAGCT |
| *IL8* | GAGTGGACCACACTGCGCCA | TCCACAACCCTCTGCACCCAGT |
| *CXCL1* | GAAAGCTTGCCTCAATCCTG | CACCAGTGAG CTTCCTCCTC |
| *ACTB* | AGAGCTACGAGCTGCCTGAC | GGATGCCACAGGACTCCA |

**Table S4.** Absolute transfection percentages (%) for each non-senescent UC-MSCs donor following treatment with the different niosomal systems or the positive control, Lipofectamine.

| Formulation | Mole ratio | DOTMA/DNA ratio (w/w) | Donor #1 (%) | Donor #2 (%) | Donor #3 (%) | Donor #4 (%) |
| --- | --- | --- | --- | --- | --- | --- |
| LPF | **-** | **-** | 21,667 ± 0,577 | 21,940 ± 0,260 | 4,437 ± 0,548 | 7,280 ± 0,560 |
| DP20CQ | **1:2:1** | **5/1** | 11,000 ± 1,000 | 16,820 ± 1,220 | 6,020 ± 0,167 | 7,747 ± 0,602 |
| DP20CQ | **1:2:1** | **10/1** | 16,417 ± 0,247 | 22,140 ± 0,460 | 4,643 ± 1,358 | 9,760 ± 0,721 |
| DP20CQ | **1:2:1** | **15/1** | 4,433 ± 0,493 | 16,890 ± 0,470 | 6,270 ± 0,513 | 10,780 ± 0,540 |
| DP20CQ | **1:2:2** | **5/1** | 12,887 ± 0,618 | 19,973 ± 0,960 | 6,110 ± 1,380 | 6,680 ± 0,280 |
| DP20CQ | **1:2:2** | **10/1** | 18,663 ± 0,552 | 21,293 ± 1,799 | 6,967 ± 0,445 | 11,500 ± 0,900 |
| DP20CQ | **1:2:2** | **15/1** | 9,767 ± 0,493 | 21,093 ± 1,335 | 7,185 ± 1,205 | 9,920 ± 0,400 |
| DP80CH | **1:2:2** | **5/1** | 7,613 ± 0,681 | 16,307 ± 0,335 | 2,960 ± 0,270 | 6,380 ± 0,540 |
| DP80CH | **1:2:2** | **10/1** | 12,133 ± 0,379 | 18,533 ± 1,161 | 4,403 ± 0,500 | 6,440 ± 0,250 |
| DP80CH | **1:2:2** | **15/1** | 11,710 ± 0,233 | 19,773 ± 1,698 | 5,030 ± 0,600 | 7,760 ± 0,520 |
| DP80CH | **1:2:4** | **5/1** | 15,283 ± 0,072 | 16,947 ± 1,960 | 3,450 ± 0,250 | 8,240 ± 0,564 |
| DP80CH | **1:2:4** | **10/1** | 15,567 ± 0,376 | 15,787 ± 1,006 | 3,017 ± 0,633 | 6,380 ± 0,260 |
| DP80CH | **1:2:4** | **15/1** | 8,577 ± 0,541 | 14,880 ± 0,080 | 4,150 ± 0,620 | 6,320 ± 0,840 |

| Formulation | Mole ratio | DOTMA/DNA ratio (w/w) | Donor #1 (%) | Donor #2 (%) | Donor #3 (%) | Donor #4 (%) |
| --- | --- | --- | --- | --- | --- | --- |
| LPF | **-** | **-** | 4,030 ± 0,154 | 22,907 ± 1,448 | 10,900 ± 0,943 | 6,253 ± 0,401 |
| DP20CQ | **1:2:1** | **5/1** | 16,300 ± 1,000 | 28,880 ± 1,634 | 9,125 ± 0,319 | 7,880 ± 1,812 |
| DP20CQ | **1:2:1** | **10/1** | 13,200 ± 1,224 | 26,627 ± 0,442 | 12,430 ± 1,473 | 10,680 ± 1,506 |
| DP20CQ | **1:2:1** | **15/1** | 17,977 ± 0,280 | 30,453 ± 1,868 | 15,065 ± 0,857 | 11,625 ± 1,785 |
| DP20CQ | **1:2:2** | **5/1** | 10,400 ± 0,529 | 17,920 ± 1,760 | 14,370 ± 0,454 | 9,107 ± 0,583 |
| DP20CQ | **1:2:2** | **10/1** | 12,980 ± 0,849 | 28,933 ± 1,661 | 16,860 ± 1,400 | 14,460 ± 1,500 |
| DP20CQ | **1:2:2** | **15/1** | 3,283 ± 0,247 | 35,227 ± 2,856 | 15,230 ± 1,835 | 17,120 ± 1,336 |
| DP80CH | **1:2:2** | **5/1** | 22,050 ± 0,522 | 21,380 ± 2,380 | 9,530 ± 1,597 | 6,347 ± 0,854 |
| DP80CH | **1:2:2** | **10/1** | 18,407 ± 1,406 | 21,853 ± 2,419 | 9,715 ± 0,415 | 6,200 ± 0,320 |
| DP80CH | **1:2:2** | **15/1** | 18,910 ± 0,443 | 16,613 ± 2,824 | 9,400 ± 1,000 | 5,840 ± 0,360 |
| DP80CH | **1:2:4** | **5/1** | 17,833 ± 0,551 | 19,733 ± 1,835 | 7,030 ± 0,035 | 7,293 ± 1,071 |
| DP80CH | **1:2:4** | **10/1** | 17,510 ± 1,054 | 17,493 ± 0,800 | 5,357 ± 0,180 | 7,960 ± 1,200 |
| DP80CH | **1:2:4** | **15/1** | 16,247 ± 0,081 | 17,520 ± 0,320 | 6,623 ± 1,617 | 4,740 ± 0,180 |

**Table S5.** Absolute transfection percentages (%) for each senescent UC-MSCs donor following treatment with the different niosomal systems or the positive control, Lipofectamine.

**Table S6.** Qualitative summary of the key parameters of the optimized niosomal formulations and DOTMA/DNA ratios (w/w), including transfection efficiency, cell viability and endosomal escape.

| Cell phenotype | Optimized formulation | DOTMA/DNA ratio (w/w) | Transfection efficiency | Cell viability | Endosomal escape |
| --- | --- | --- | --- | --- | --- |
| Non-senescent | **DP20CQ 1:2:2** | **10/1** | +++++ | +++++ | +++++ |
|  | **DP20CQ 1:2:1** | **10/1** | ++++ | +++++ | +++ |
|  | **DP20CQ 1:2:1** | **15/1** | ++ | +++++ | ++ |
| Senescent | **DP20CQ 1:2:2** | **10/1** | ++++ | +++++ | +++++ |
|  | **DP20CQ 1:2:1** | **10/1** | +++++ | +++++ | ++ |
|  | **DP20CQ 1:2:1** | **15/1** | +++ | +++++ | + |
